# Altered growth and death in dilution-based viral predation assays

**DOI:** 10.1371/journal.pone.0288114

**Published:** 2023-07-07

**Authors:** Ben Knowles, Juan A. Bonachela, Nick Cieslik, Alice Della Penna, Ben Diaz, Nick Baetge, Micheal J. Behrenfeld, Karen Naumovitz, Emmanuel Boss, Jason R. Graff, Kimberly H. Halsey, Liti Haramaty, Lee Karp-Boss, Kay D. Bidle

**Affiliations:** 1 Department of Marine and Coastal Science, Rutgers University, New Brunswick, New Jersey, United States of America; 2 Department of Ecology and Evolutionary Biology, University of California, Los Angeles, Los Angeles, California, United States of America; 3 California NanoSystems Institute, University of California, Los Angeles, Los Angeles, California, United States of America; 4 Institute for Quantitative and Computational Biosciences, University of California, Los Angeles, Los Angeles, California, United States of America; 5 Institute of the Environment and Sustainability, University of California, Los Angeles, Los Angeles, California, United States of America; 6 Department of Ecology, Evolution, and Natural Resources, Rutgers University, New Brunswick, New Jersey, United States of America; 7 School of Biological Sciences, University of Auckland, Auckland, New Zealand; 8 Institute of Marine Science, University of Auckland, Auckland, New Zealand; 9 Department of Ecology, Evolution, and Marine Biology, University of California Santa Barbara, Santa Barbara, California, United States of America; 10 Department of Botany and Plant Pathology, Oregon State University, Corvallis, Oregon, United States of America; 11 School of Marine Sciences, University of Maine, Orono, Maine, United States of America; 12 Department of Microbiology, Oregon State University, Corvallis, Oregon, United States of America; Second Institute of Oceanography Ministry of Natural Resources, CHINA

## Abstract

Viral lysis of phytoplankton is one of the most common forms of death on Earth. Building on an assay used extensively to assess rates of phytoplankton loss to predation by grazers, lysis rates are increasingly quantified through dilution-based techniques. In this approach, dilution of viruses and hosts are expected to reduce infection rates and thus increase host net growth rates (i.e., accumulation rates). The difference between diluted and undiluted host growth rates is interpreted as a measurable proxy for the rate of viral lytic death. These assays are usually conducted in volumes ≥ 1 L. To increase throughput, we implemented a miniaturized, high-throughput, high-replication, flow cytometric microplate dilution assay to measure viral lysis in environmental samples sourced from a suburban pond and the North Atlantic Ocean. The most notable outcome we observed was a decline in phytoplankton densities that was exacerbated by dilution, instead of the increased growth rates expected from lowered virus-phytoplankton encounters. We sought to explain this counterintuitive outcome using theoretical, environmental, and experimental analyses. Our study shows that, while die-offs could be partly explained by a ‘plate effect’ due to small incubation volumes and cells adhering to walls, the declines in phytoplankton densities are not volume-dependent. Rather, they are driven by many density- and physiology-dependent effects of dilution on predation pressure, nutrient limitation, and growth, all of which violate the original assumptions of dilution assays. As these effects are volume-independent, these processes likely occur in all dilution assays that our analyses show to be remarkably sensitive to dilution-altered phytoplankton growth and insensitive to actual predation pressure. Incorporating altered growth as well as predation, we present a logical framework that categorizes locations by the relative dominance of these mechanisms, with general applicability to dilution-based assays.

## Introduction

Viral lysis of microbial hosts is one of the most common forms of death on Earth. It sculpts the evolution and composition of host communities from molecular to global scales by selecting against infection-sensitive lineages and driving boom-bust cycles of proliferating hosts [1–5]. Lysis further underpins global ecosystem processes like biogeochemical cycling and food web function by liberating cellular metabolites from phytoplankton that can then be metabolised by heterotrophic bacteria via the Viral Shunt [6–9], possibly after being displaced to pelagic depths by the Viral Shuttle [10–14], that can then be tranfered to higher trophic levels via grazing in the Microbial Loop [15–18]. In addition to viral lysis, cellular debris can also be made available for bacterial metabolism by inefficient grazer feeding [9, 19, 20], and grazing is a major means of channeling microbial biomass to higher trophic levels [15, 21, 22].

Lysis and grazing rates are commonly assessed using dilution-based approaches. The well-established ‘Dilution Assay’, originally used to quantify grazing rates on phytoplankton [23–25], has increasingly been extended to assess lysis rates in field settings (*e*.*g*., [26–34], reviewed in [35]). Treating viruses as analogous to grazers, this approach reduces virus and host densities by dilution to lower virus-host encounter and infection rates. Increases in apparent growth rates of phytoplankton (AGR; change in phytoplankton cell concentration over 24 hours; *i*.*e*., net accumulation rate) with increased dilution are interpreted as a measure of grazing and viral lysis in the undiluted samples.

In laboratory settings, viral infections are commonly quantified using a high-throughput dilution method known as the ‘Most Probable Number’ (MPN) assay, which involves applying a dilution series of viral lysate to algal cultures in 96-well plates with ~ 200 μL incubations and monitoring the decline of cells over weeks as evidence of infection [36–41]. Both the MPN and dilution assays rely on the assumption that dilution-altered net growth rates serve as a proxy for predation rates. In particular, they assume that dilution only affects virus-host encounters and predation rates but does not affect host physiology.

In this study, we combined the viral form of the well-established and broadly-used Dilution Assay with the high-throughput approach of plate-based MPN assays using high precision flow cytometry (*i*.*e*. cell counts down to < 10^2^ per sample) to conduct high-throughput, high-replication 96-well plate dilution assays [26, 31, 33, 34, 42–44]. This approach assumed (*i*) that the growth of natural phytoplankton populations in microtiter plates would mirror those seen in traditional experiments with larger incubation volumes and (*ii*) that the established assumptions of the Dilution Assay hold.

We implemented this approach in 18 experiments at a freshwater pond in the Northeast United States and marine sites in the Sargasso Sea/Gulf Stream, sub-tropical North Atlantic, and temperate North Atlantic to investigate the relationship between lysis rates and environmental conditions. Prior work during the late Spring shows that while there were measurable grazing rates at the North Atlantic sites, with grazing highest on cyanobacteria and lowest on pico-eukaryotes, only minimal viral lysis was observed [34]. During the early Spring cruise on which we conducted this work, phytoplankton communities were characterized as cyanobacterial-dominated based on pigment [45] and ribotyping [46] analyses. Phytoplankton communities were also experiencing high accumulation rates, low stress and death rates, and low extracellular viral concentrations during the formation of the seasonal North Atlantic spring bloom [47, 48].

However, our experiments showed unexpected and counter-intuitive results that we have sought to explain. Contrary to published dilution experiments, the most notable outcome of our study was a decline in phytoplankton densities that was exacerbated by dilution, which would usually be interpreted as ‘negative’ lysis rates. In other words, phytoplankton growth rates were lower in more diluted treatments than in less diluted treatments, yielding negative slopes in AGRs across dilution levels. This was consistent with paired traditional 1 L dilution experiments done at the same time and for the same sites, but it was contrary to the predicted increases in growth due to reduced virus-host encounter and infection rates.

We conducted environmental, theoretical, and culture-based analyses to attempt to understand why our results differed from the expectations of standard dilution assays. Our analyses showed that various growth and loss mechanisms operate in dilution-based virus-host lysis assays and that the relative influence of these mechanisms can be used to classify locations as dominated by either predation, nutrient limitation, or dilution-induced lagged phytoplankton growth.

## Results and discussion

### Initial assumptions and expectations

**Fig 1:**
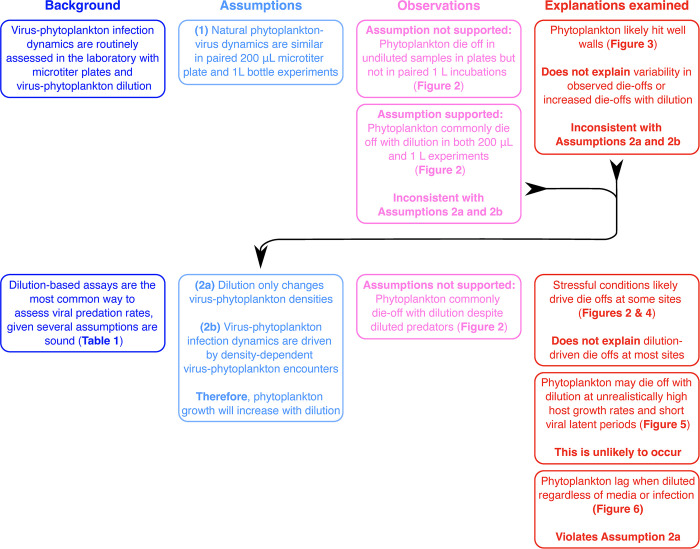
The logical structure of the project. We combined existing laboratory microtiter plate and field dilutions-based assays (“Background’, blue text and boxes) to make a new high-throughput viral predation assay, based on the assumptions based on established approaches (‘Assumptions’; light blue) that were either met or not (‘Observations’; pink). Violated assumptions then led us to experimentally and theoretically examine a range of possible explanations (‘Explanations examined’; red).

**Fig 2:**
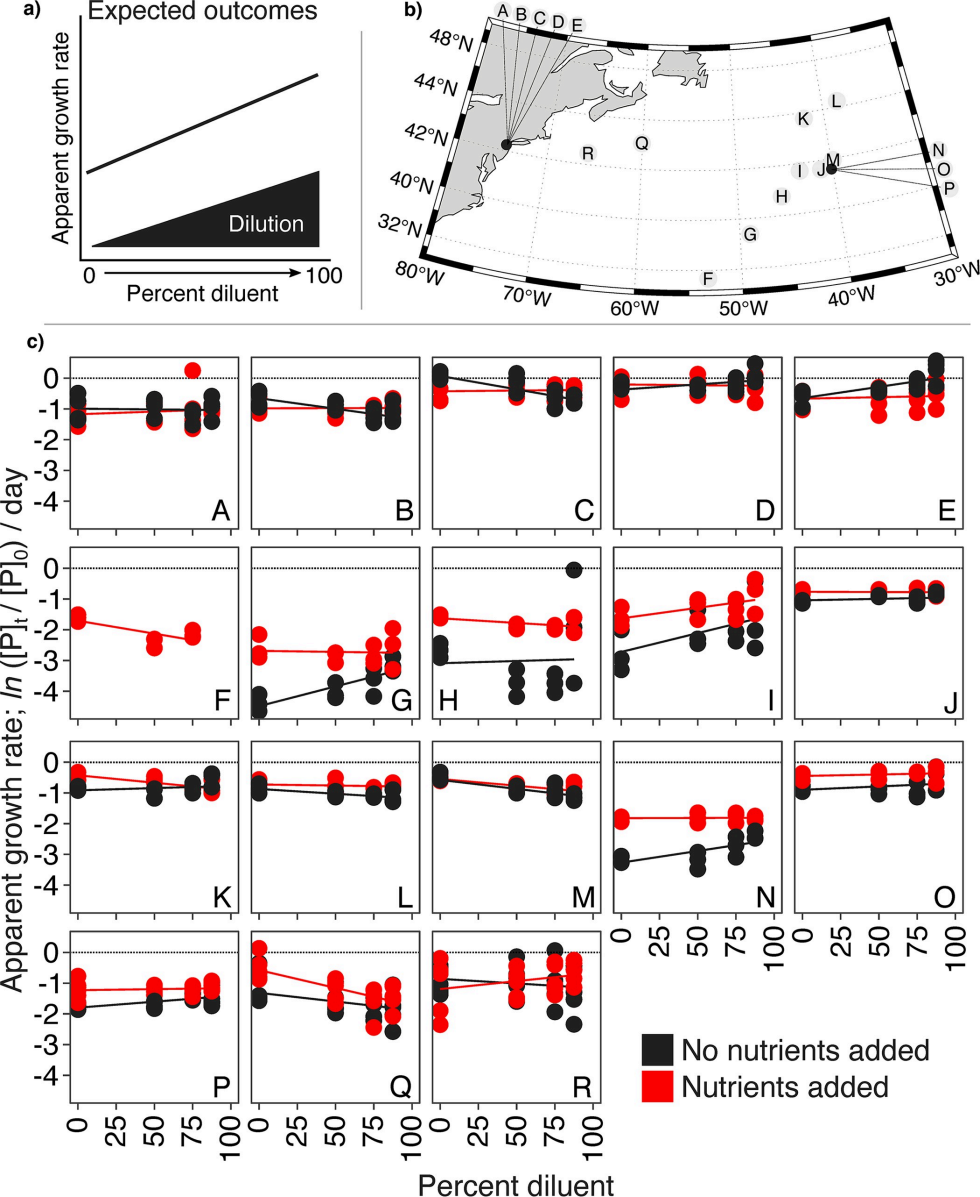
Expected outcomes, field site locations, and empirical data from microtiter dilution assays. (**a**) Idealized expected results from dilution assays where phytoplankton apparent growth rates increase as viruses are diluted. (**b**) The distribution of experiments across New Jersey, the Sargasso Sea/Gulf Stream, sub-tropical North Atlantic, and temperate North Atlantic. (**c**) AGRs across dilution in each experiment are shown with or without nutrients (red and black data points and linear lines of best fit, respectively). n = 4 for Experiments A–E (each panel shows 32 data points), n = 3 for experiments F–O (each panel shows 24 data points), and n = 6 for experiments P and Q (each panel shows 48 data points). Apparent growth rates are the change in phytoplankton densities over 24 hours; AGR = *ln* (P_t_/P_0_) / day, where P_t_ and P_0_ are the number of phytoplankton cells per mL at the end and start of the incubation period, respectively. Maps in panel (**b**) were made using the m_maps package in Matlab [68] with publicly-available chlorophyll data from the NASA Ocean Biology Processing Group (https://earthdata.nasa.gov/).

Our study was based on two *a priori* assumptions (**Fig 1**). First, it assumed that single-strain, culture-based assays (like MPNs; [36–41]) with low incubation volumes can be extended to mixed communities and larger volumes. If true, this would allow measurement of predation dynamics in a low incubation-volume, high-throughput manner in 96-well microtiter plates. Second, it assumed that dilution only affects host and virus densities and does not impact cellular physiology or community function. This is a commonly held assumption that underpins dilution-based approaches in general (**Table 1**) [23, 26, 49]. If true, the altered net growth and phytoplankton accumulation rates observed in the assay could be ascribed to changes in viral predation rates. (**Table 1, Figs 1 and 2A**).

**Table 1:** Assumptions required to obtain predation rates using dilution techniques. Assumptions we adopted when designing our experimental approach. These assumptions are either explicitly stated in the original papers (*e*.*g*., [23, 26, 49]) or mathematically required (‘Assumption…’) for the dilution approach to be soundly used. Each assumption is also shown with its corresponding outcomes (‘Entails…’) and necessary preconditions (‘Valid…’).

Assumption…	Entails…	Valid…
**1**: Phytoplankton population grows exponentially in the absence of predation (see Methods, Eq (1))	Growth and mortality rates do not change during incubation (see below)	When nutrient availability or top-down pressure do act on or limit phytoplankton growth
**2**: Natural mortality is negligible with respect to top-down regulation (Eq (3))	Cells only die due to grazing or viral lysis	If resources are high enough to prevent starvation, and incubation time is shorter than the typical cell lifetime
**3**: Grazing (viral) mortality rates are proportional to the density of the grazer (viral) population (Eqs (5) and (7))	Grazers (viruses) and phytoplankton cells encounter each other at random	In well-mixed environments and if the digestion time (latent period) are short
**4**: Phytoplankton growth rate does not change with time	Nutrient availability does not affect the growth rate	When nutrient availability does not limit phytoplankton growth
**5**: Lysis rates do not change with time	Virus density does not change with time	When contact rates with predators or the predator-prey ratio remains constant during incubation time.

**Fig 3:**
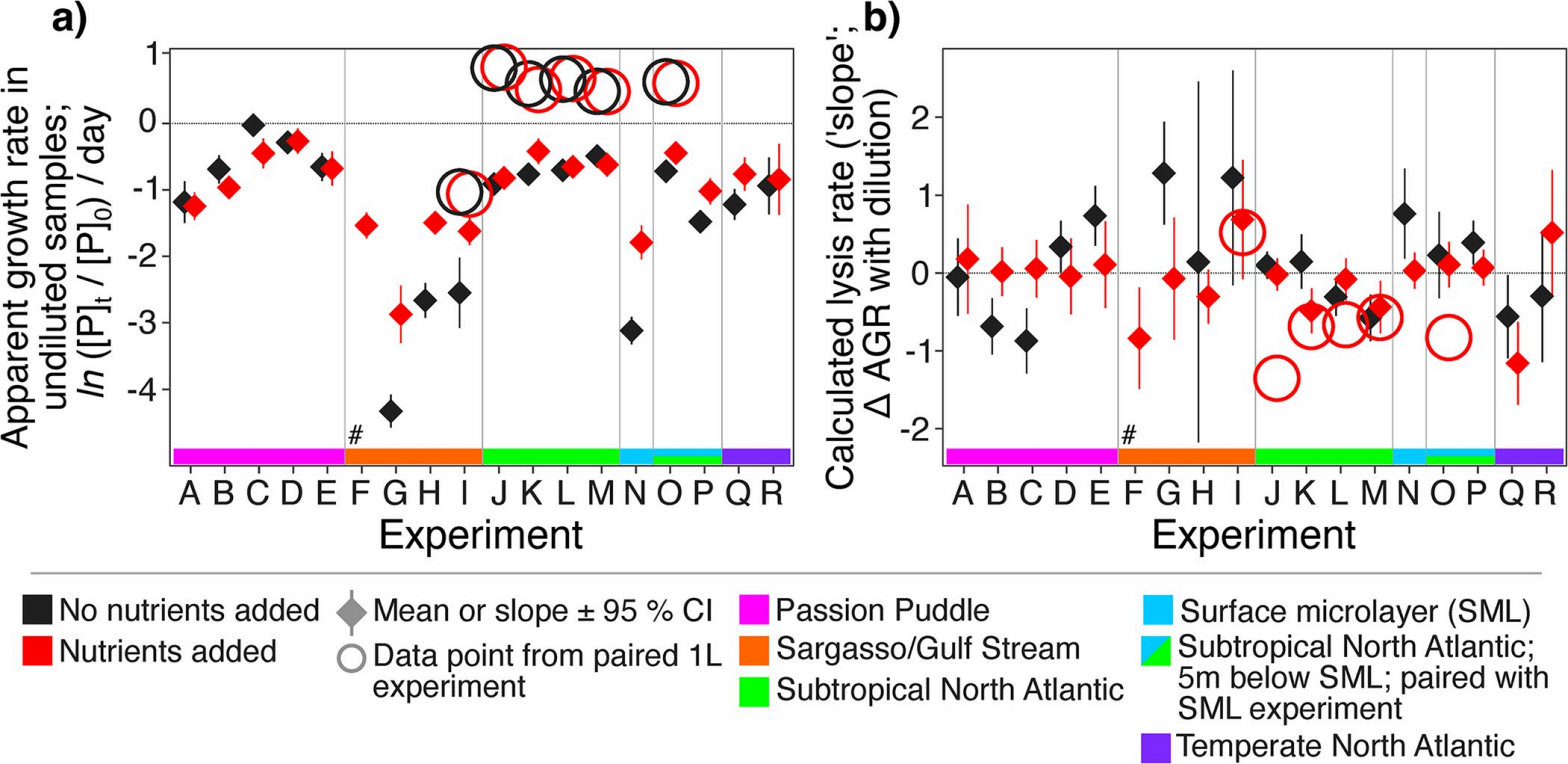
Apparent growth rates in undiluted samples and lysis rates in microtiter dilution assays. (**a**) Apparent growth rates in undiluted samples (the 0% diluent values in **Fig 2C**, shown ± 95% confidence intervals of the mean; AGRs are the change in phytoplankton densities over 24 hours; AGR = *ln* (P_t_/P_0_) / day, where P_t_ and P_0_ are the number of phytoplankton cells per mL at the end and start of the incubation period, respectively). (**b**) Calculated lysis rates (slopes; the change in AGR across dilutions shown ± 95% confidence intervals of the slope). Both (**a**) and (**b**) show outcomes of larger 1 L volume incubations that shared the same diluents and site water as the microtiter experiments (circles). Experiments are color-coded by site water mass and divided by vertical lines. Data points are colored by nutrient addition with and without nutrients added (red and black data points and confidence intervals, respectively).

Our experimental observations across an urban pond in New Jersey and the North Atlantic Ocean (**Fig 2B, S1 Table in S1 File**), though, suggest that neither of the above assumptions are satisfied (**Figs 1 and 2C**). First, we observed widespread negative growth rates across all cell sizes in our microtiter plates and in undiluted samples in particular, which was not seen in the paired 1 L incubations (**Figs 2C and 3A, S2 Table in S1 File; S1 Fig in S1 File**). This suggests that the ‘die-offs’ could have been incubation volume-dependent, violating our first assumption (**Fig 1**). Second, growth rates changed inconsistently and contrary to dilution level, at odds with the expectation of our second assumption where increasing dilution would increase net growth by reducing virus-phytoplankton encounters (**Fig 3B)** [23, 26, 49]. Importantly, the fact that phytoplankton die-offs were also observed in dilutions in paired 1 L incubations suggests these effects are not an artifact of incubation volume but are a general characteristic of dilution-based approaches. Because dilution approaches are so integral to measuring loss and predation rates in the environment (*e*.*g*., the dilution assay has been used to generate > 788 grazing rate and phytoplankton growth rate datasets; [25]), we sought a mechanistic understanding of why the assay assumptions were violated.

### Methodological concerns with low incubation volumes

We hypothesized that two key factors may drive the high mortality rates we observed in the low-volume experiments and not in the 1 L incubations: sinking and adhering to well walls [50]. Since the experimental ‘water column’ height of seawater in each microtiter well was ~ 5 mm compared to 100 to 200 mm in 1 L incubations, phytoplankton sinking to the bottom of the wells might underpin the negative growth rates we observed in our plates. Cells of similar size to those in our samples can sink 100–300 mm in 24 hours [51, 52]. These rates imply that cells with no vertical mobility could sink to the bottom of the wells within a few hours without active resuspension. While this phenomenon might be partly responsible for the observed mortality rates only in the low-volume incubation experiments, it does not explain why such mortality is exacerbated by dilution.

**Fig 4:**
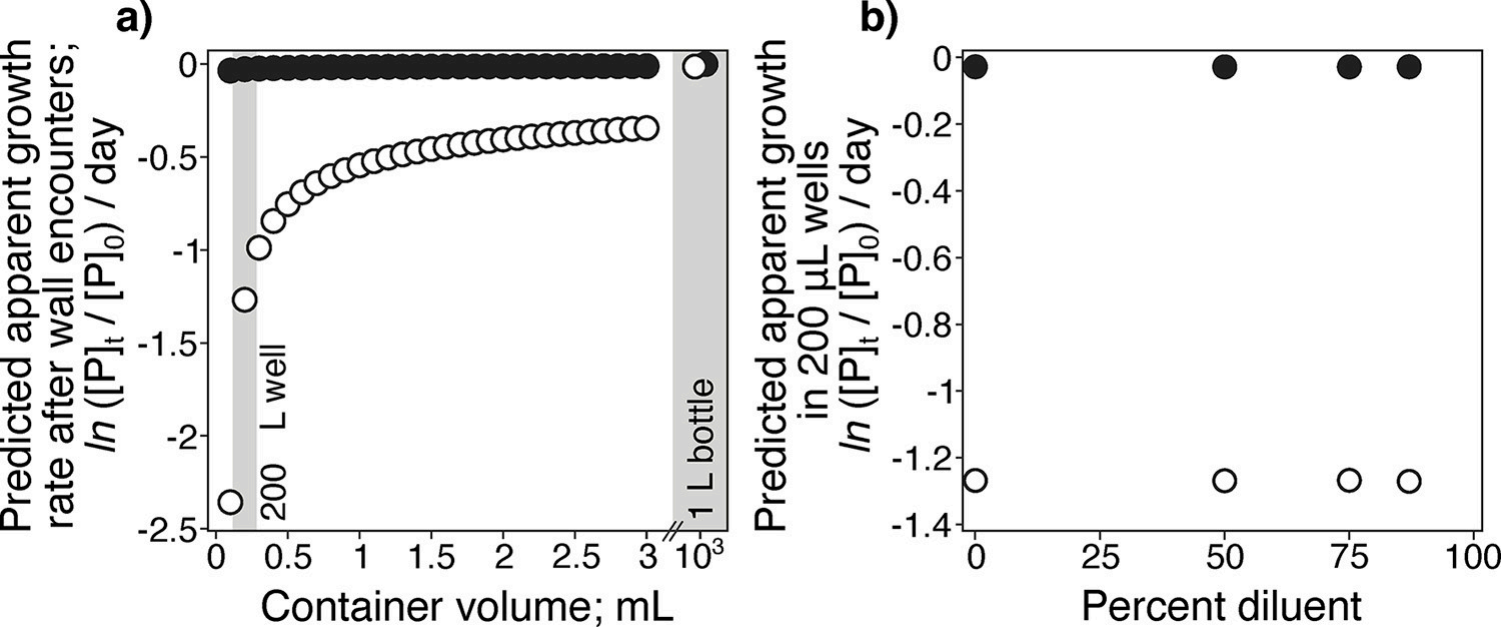
Small incubation volumes alone are not responsible for highly negative apparent growth rates or decreasing phytoplankton growth with dilution. (**a**) Spatially-explicit modeling of swimming and non-motile phytoplankton (open and black circle, respectively) calculated apparent growth rates after accommodating wall encounters in different-sized containers. (**b**) The lack of effect of dilution on the apparent growth rate of swimming and non-motile phytoplankton in 200 μL incubations (predicted lysis rate = 0). Non-motile random Brownian diffusion and random swimming were modeled with diffusion coefficients of 0.1 and 10 μm^2^ per second, respectively. Note the broken x-axis in (**b**) to compare microtiter plate and 1000 mL (*i*.*e*., 1L) incubations.

It is also possible that phytoplankton cells might have stuck to the microtiter plate walls during our experiments, preventing them from being counted at the final timepoint. To determine the effects of incubation volume and wall encounters, we modeled the movement of cells colliding with walls in different-sized containers to possibly explain the negative apparent growth rates (AGRs) in undiluted samples (**Figs 3A and 4**), allowing cells to move with effective diffusion coefficients of 0.1 μm^2^ per second (diffusion only) and 10 μm^2^ per second (diffusion and motility), respectively [20, 53]. Under both low and high diffusion, cells were predicted to encounter walls more frequently in the 200 μL wells than in 1 L incubations (**Fig 4A**). With only diffusion, the cells are predicted to encounter walls infrequently, reducing daily AGRs by only ~ 0.05, regardless of incubation volume (**Fig 4A**). However, with active motility, wall encounters reduced AGRs by ~ 1.25 in the 200 μL wells (**Fig 4B**), similar to the AGR reductions observed in many experiments (**Fig 3A**). Yet, AGRs did not change across dilution levels in response to wall encounters (**Fig 4B**). Thus, the negative slopes (*i*.*e*., negative lysis rates) we observed in our experiments were not explained by wall encounters. We therefore considered the intrinsic effects of the dilution method itself rather than the incubation volume to explain our observations.

### Effect of stress on growth and lysis

The most negative apparent growth rates in the undiluted samples were observed in the Sargasso Sea and Gulf Stream (Experiments F-I) and surface microlayer samples (Experiment N; **Fig 2C**). The surface microlayer is known to be a stressful environment [54], and samples in Experiment N exhibited stress signatures of low chlorophyll:biomass ratios compared to ratios observed in samples collected at 5 m depth at the same site and used for Experiments O and P (**S2 Fig in S1 File)** [55]. This finding suggests that cell physiology and stress-related mechanisms like reactive oxygen stress or nutrient limitation are at play in the surface microlayer [48, 54] that might have been missed if not using flow cytometry to quantify loss rates. Furthermore, experiments G-I and N showed markedly positive impacts of nutrient addition on growth. In these samples, undiluted die-offs are ameliorated and slopes commonly changed signs (**Fig 3B**; *i*.*e*., lysis rates changed signs) when nutrients were added. Hence, what appears as evidence of strong predation (*i*.*e*., positive slopes) can reflect nutrient limitation and stress, providing a metric observable through the dilution assay by comparing the undiluted AGRs to treatments where nutrients are added or not.

### Theoretical generation of negative lysis rates

**Fig 5:**
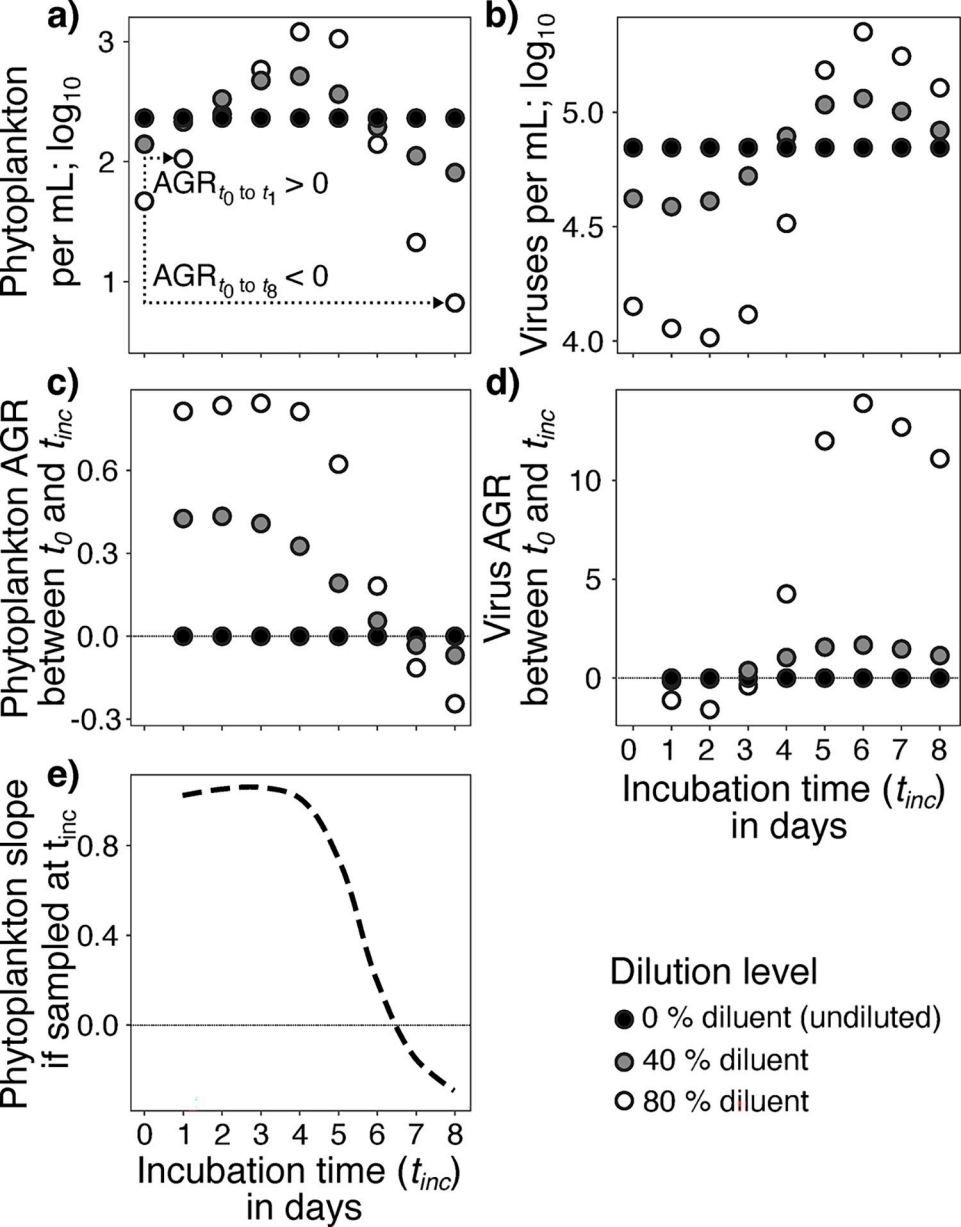
Viral reproduction can yield positive to negative slopes over time in the dilution assay. Model predictions of (**a**) phytoplankton and (**b**) viral densities over time (incubation time; *t*_*inc*_) across a range of dilutions (80%, 40%, and 0% diluent treatments are shown as black, grey, and open white circles, respectively). Changing predator densities allows growth rates to change from positive to negative in any dilution (see dashed lines in panel (**a**) for how AGRs can change signs if measured between *t*_0_ and *t*_1_ *vs*. *t*_0_ and *t*_8_). Panels (**c**) and (**d**) show changes in AGR values over time for each dilution of phytoplankton and viruses, respectively, which then (**e**) results in changing lysis rates. This model was parameterized by the *E*. *huxleyi-EhV* system [62].

We next asked whether negative lysis rates (*i*.*e*., lower phytoplankton AGRs in diluted samples, yielding negative slopes) could be explained by top-down regulation in experiments. We implemented a virus-host theoretical model that mirrored the assumptions and top-down logic of the dilution assay (**Fig 5, Table 1)** [23, 26]. Following phytoplankton and viral densities (**Figs 5A and 5B; S3 Fig in S1 File**), the model indicates that each dilution factor has unique dynamics (**Fig 5C and 5D**). Diluted phytoplankton populations initially show rapid growth (*e*.*g*., AGRs measured between *t*_*0*_ and an incubation time (*t*_*inc*_) < 6 days in a model parameterized for the phytoplankton *Emiliania huxelyi* and its viruses, EhVs; **Fig 5C**). However, the AGR turns negative–*i*.*e*., phytoplankton die-off–if measured over longer timespans like 8–10 days because of viral lysis from increased virus-host encounter rates and infection (**Fig 5D**). In other words, the relationship between AGRs observed in each dilution factor changes over time. This result makes estimating lysis rates–the slope of the line across AGRs in each dilution–a ‘moving target’ that depends on the incubation time (*t*_*inc*_; **Fig 5E**). This effect is exacerbated in systems with higher virus:host ratios (**S4 Fig in S1 File**). However, changing viral titres is unlikely to yield the observed negative responses to dilution (*i*.*e*., negative lysis rates) because dilution assays, since their inception, are run for only 1 day to avoid such an outcome (e.g., [23, 26]). This is reinforced by latent periods of marine viruses are being modeled to average ~ 24 hours, albeit with a range from ~ 2 to 48 hours, similar to the incubation length of the assay [56].

Given that we had broadly explored whether predator-prey dynamics could have realistically yielded suppressed host growth with dilution in our modeling (*e*.*g*., **Fig 5, S3 and S4 Figs in S1 File**; see S1 File for a detailed theoretical consideration of changing predation pressure and its implications for the dilution assay) and not found outcomes consistent with our experiments, we further examined other possible mechanisms not considered in the assay to explain and interpret how phytoplankton AGRs could decline with dilution.

### The effect of trophic cascades and phytotoxins

**Fig 6:**
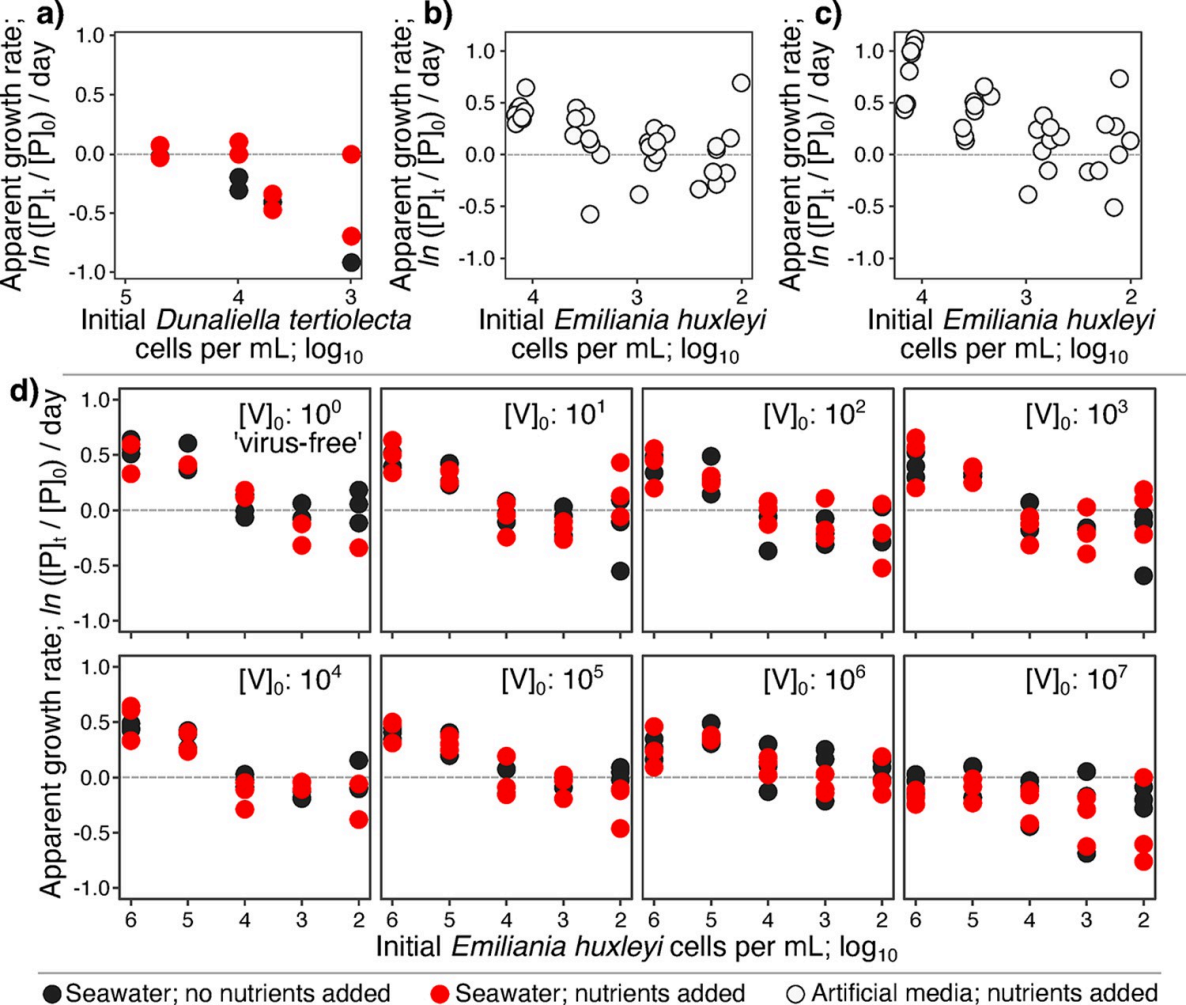
Dilution-induced lag violates a fundamental assumption of the dilution assay by modifying phytoplankton growth. (**a**) *Dunaliella tertiolecta* dilution series in cultures without predators show negative slopes due to dilution-induced lagged growth. *Emiliania huxelyi* suspended in artificial media devoid of inhibitors or predators in (**b**) 200 μL microtiter plate and (**c**) 40 mL flask incubations also show negative slopes (*i*.*e*., negative ‘lysis rates). (**d**) *E*. *huxelyi* cultures with initial viral densities of 10^0^ ‘extinct’ - 10^7^ viruses per mL also show negative slopes, with viral predation lowering the AGRs in cultures only at viral and phytoplankton densities (> 10^6^ viruses and cells per mL; c.f., viral and *E*. *huxleyi* densities of < 10^5^ and 10^4^ per mL in nature; [62]). Data points are colored by nutrient addition with and without nutrients added (red and black data points, respectively). Apparent growth rates are the change in phytoplankton densities over 24 hours; AGR = *ln* (P_t_/P_0_) / day, where P_t_ and P_0_ are the number of phytoplankton cells per mL at the end and start of the incubation period, respectively.

Suppressed phytoplankton growth rates at high dilution in incubations are often thought to arise from diluent-borne toxins inhibiting phytoplankton growth [57, 58] or from trophic cascades triggered by grazing [59]. It is difficult to distinguish the effects of phytotoxins in the diluent from the direct effects of dilution in natural samples because they are conflated. To extricate these factors, we diluted axenic (and thus predator-free) cultures of *Dunaliella tertiolecta*, a unicellular alga commonly used to model responses to stress [60], into 0.02 μm-filtered diluent during experiment R. If phytoplankton growth is suppressed by inhibitors in the diluent, suppression should occur in all treatments. However, this was not observed. Instead, *D*. *tertiolecta* growth rates responded positively to dilution and nutrient addition while the nutrient-deplete natural community and the *D*. *tertiolecta* both responded negatively to dilution without nutrient additions (**Fig 6A**). If negative responses to dilution are driven by trophic cascades, we should not see them in the predator-free *D*. *tertiolecta* treatment. The observed negative responses to dilution in this treatment revealed that trophic cascades are not driving the observed outcomes in our experiments (**Fig 6A**). Taken together, there must be other factors involved, such as dilution-induced changes to phytoplankton growth rates [61].

### Dilution-induced lagged phytoplankton growth

The direct negative effects of dilution on phytoplankton growth were confirmed when we repeated our experiments in the laboratory with *E*. *huxleyi* cultures in the absence of predators [62]. Unlike in the *D*. *tertiolecta* experiments, the artificial and defined media used in the lab experiments with *E*. *huxleyi* were known to be free of growth inhibitors (*i*.*e*., these experiments were free of both trophic cascades and phytotoxins; **Fig 6B–6D**). These experiments, which were recapitulated by theoretical modeling in the presence of dilution-induced lagged growth and the absence of predators (**S5 Fig in S1 File**), showed that dilution alone can generate negative slopes *via* lagged phytoplankton growth [61]. This innate lag is also independent of incubation volume. Indeed, negative relationships between apparent growth rate and dilution were observed in microtiter plate cultures (**Fig 6B**) as well as in paired 40 mL flasks (**Fig 6C**). In summary, increased phytoplankton die-offs can be observed as an intrinsic outcome of dilution when dilution-induced lag suppresses phytoplankton growth below replacement from death processes [61]. Extrinsic mechanisms like predation, inhibitors, and trophic cascades are not required to explain this response.

### Dilution-induced lagged phytoplankton growth vs. predation

To disentangle the effects of dilution-induced lagged phytoplankton growth from the predator-free experiments and predatory death processes, we conducted experiments with a range of abundances of *E*. *huxleyi* cells and viruses (**Fig 6D***; E*. *huxelyi* virus; EhV; latent period ≥ 4.5 hours; [63]). Once again, increasingly negative AGRs were observed with dilution, generating negative slopes in all treatments (**Fig 6D**). The main effect of altered viral predation pressure was not an increase in AGRs when alleviated by dilution, but rather, suppression of host AGRs in concentrated host cultures at high viral titers (**Fig 6D** panels with initial viral densities [V]_0_ of greater than 10^5^ viruses per mL). This occurred only when viral and phytoplankton densities exceeded those observed in nature (~ 10^5^ EhVs and ~ 10^3^ *E*. *huxleyi* per mL; [62, 64]). These experiments show that the dilution assay is remarkably sensitive to dilution-mediated lagged growth of phytoplankton populations and insensitive to actual viral predation pressure, especially at natural densities (**Fig 6D**).

### Predation-dominated, nutrient-limited, and lag-sensitive sites

**Fig 7:**
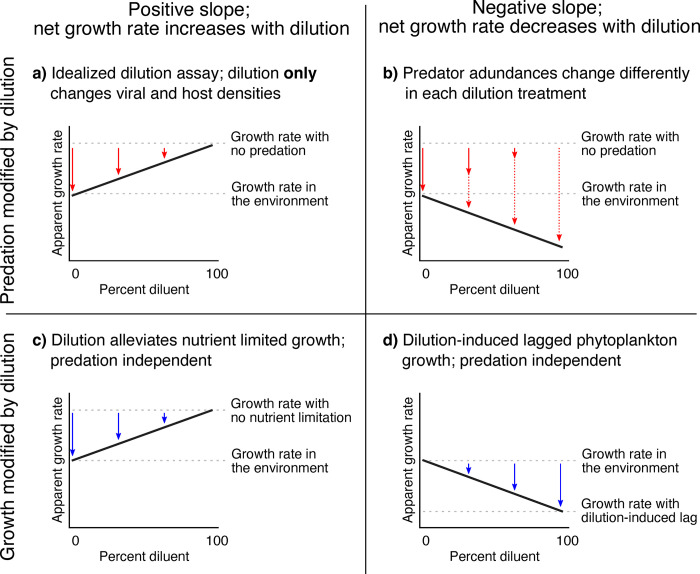
Schematic of growth and death processes operating within the dilution assay that can result in positive or negative slopes. Predation (red arrows) being reduced by dilution can lead to (**a**) positive slopes under the idealized dilution assay dynamics or (**b**) negative slopes if changing predation pressure occurs (solid and dotted arrows show predation pressure at the start and end of incubations, respectively). Factors suppressing growth (blue arrows) can lead to (**c**) positive slopes by dilution alleviating extreme nutrient limitation or (**d**) negative slopes by inducing lagged phytoplankton growth with dilution. Processes are not mutually exclusive in any given sample, and slopes observed for any sample may reflect the balance of these processes all operating.

**Fig 8:**
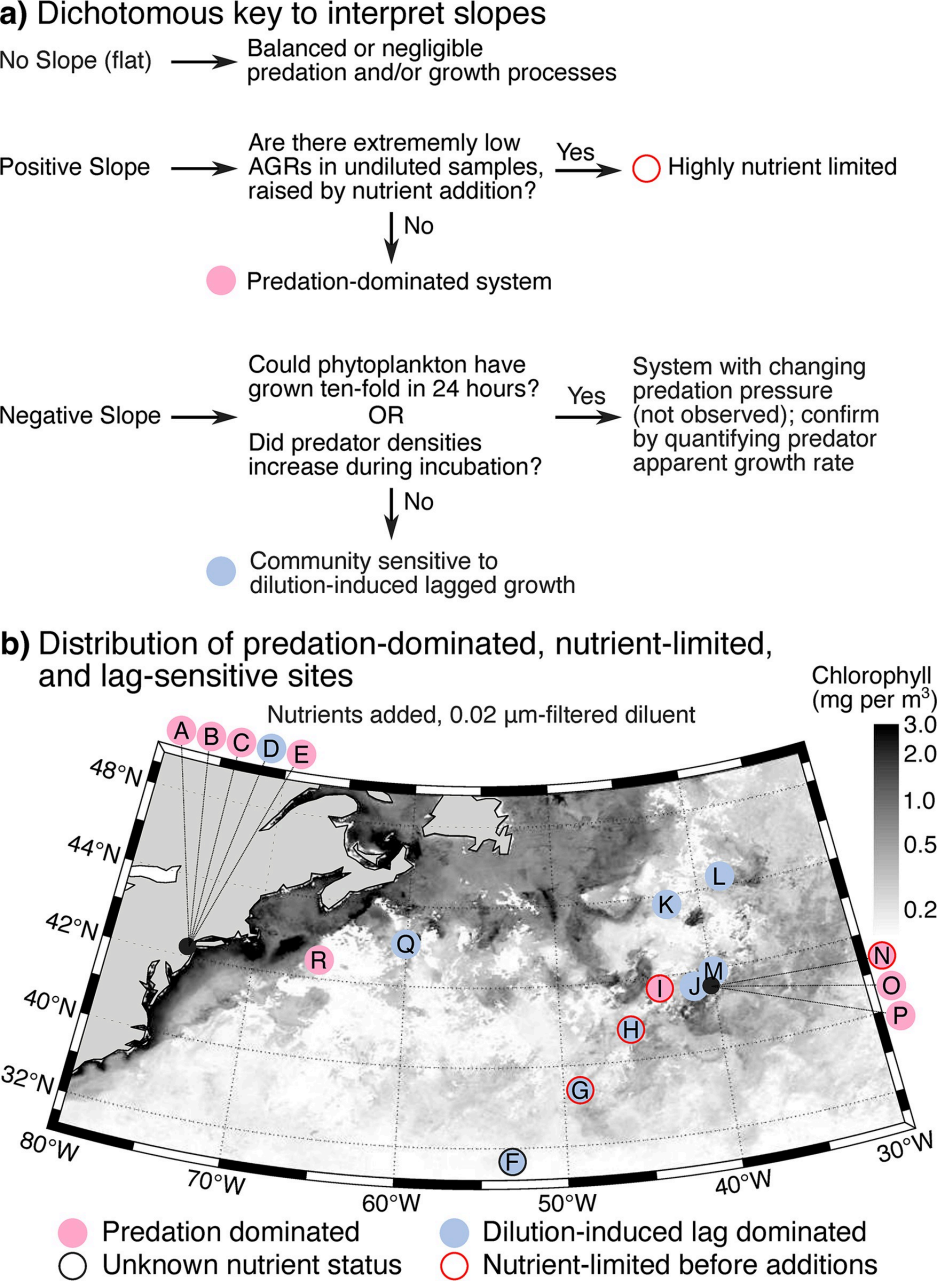
Logically binning sites into predation-dominated, high-tempo with predator growth, nutrient-limited, and lag-sensitive. (**a**) A dichotomous key to categorize sites as dominated by predation (pink circles), subject to changing predation pressure (deemed unlikely to occur in our experiments), nutrient-limited (red rings), or dilution-induced lagged phytoplankton growth (blue circles) based on whether growth rate increases or declines with dilution (positive or negative slopes, respectively) and response to nutrient addition. (**b**) The field study locations classified using this logic. Maps in panel (**b**) were made using the m_maps package in Matlab [68] with publicly-available chlorophyll data from the NASA Ocean Biology Processing Group (https://earthdata.nasa.gov/). Grey shading shows surface chlorophyll concentrations (mg per m^3^) in the North Atlantic during the sampling period.

Altogether, we conclude that many predation and growth mechanisms operate within dilution assays. Specifically, AGRs–net growth rates that are the difference between death and growth terms–reflect a balance of ‘death-side’ established dilution-assay dynamics like predation and dynamic predation rates (**Fig 7A and 7B**), as well as ‘growth-side’ nutrient limitation and dilution-induced lagged growth (**Fig 7C and 7D**). This opens the possibility that we can derive more information than just predation processes from dilution-based predation assays by considering each sampling site in terms of ‘death-side’ and ‘growth-side’ initial conditions (**Fig 8A**). To start, Passion Puddle experiments (A-E) were characterized by predation-dominance and lack of nutrient limitation, consistent with this water body being a eutrophic, suburban pond with high biomass and high predation rates (**Fig 8B**; https://www.inaturalist.org/projects/the-passion-puddle-project). Only the oligotrophic Sargasso Sea and Gulf Stream sites showed patterns consistent with nutrient limitation (Experiments F-I in **Fig 2C**) [65, 66]. Lag sensitivity is also a common trait for the majority of North Atlantic sites in sub-tropical waters, as revealed by declining growth rates with dilution (Experiments J-M; **Fig 8B**). The few samples in this area that are predation-dominated come from a site that was experiencing the die-off of a phytoplankton bloom (Experiments O-P; [47, 48]), consistent with our classification (**Fig 8B**). Altogether, our classification is consistent with existing knowledge of our experimental sites, showing that the dilution assay can provide much richer insight on plankton ecosystems than currently thought.

## Conclusions

Estimates of predation pressure, by grazers or viruses, are commonly founded on dilution assays. Whether addressing grazing or viral predation, the dilution approach shares common logic, strengths, and challenges regardless of the predator considered. These assays assume that dilution changes only the densities of hosts and predators without affecting trophic dynamics or host physiology and predator fecundity. Beyond showing that microtiter plates are a poor format for dilution assays, our analysis shows that these assays are highly sensitive to the physiological state of cells at experimental sites and to the pervasive physiological effects of experimental dilution on those cells. We would not have seen these effects if we had excluded our counter-intuitive results (i.e., apparently negative lysis rates), as has been a common practice in the field. Looking forward, our findings make a strong case for reporting all data from dilutions experiments and for incorporating measures like predator abundances and nutrient availibility into the assay. Our findings undercut the utility of dilution-based approaches for quantifying predation rates alone by showing the dilution assay to remarkably sensitive to dilution-altered phytoplankton and insensitive to actual predation pressure. But this insight also shows that the assay can be broadened to provide a more holistic understanding of ecological processes by considering physiology-dependent as well as density-dependent growth and death processes.

## Methods

### Initial assumptions

Our study was based on two reasonable *a priori* assumptions (**Fig 1**): (*i*) that environmental phytoplankton communities will not be negatively affected by putting them in low-volume microtiter plates, and (*ii*) that dilution of these communities will only modify the rates of phytoplankton encountering predators/viruses and not modify cellular physiology or predator reproduction. If these assumptions were sound, it would allow us to conduct high-throughput dilution-based predation/lysis assays ascribing elevated phytoplankton growth in diluted samples to alleviated predation pressure, a proxy measure of lysis rates (e.g., [23, 26, 49]; **Table 1)**.

### Sampling and processing

Freshwater samples were collected from a suburban pond in New Brunswick, NJ, U.S.A. (‘Passion Puddle’) between February 8^th^ and March 8^th^, 2018 by gently submerging and filling an autoclaved 500 mL glass bottle and sealing it with ~ 50 mL headspace (**S1 Table in S1 File**; Experiments A -E). Water was processed within an hour at 18°C and microtitre plates were then incubated for 24 hours at either 4 or 15°C (**S1 Table in S1 File**; incubation temperatures were on average 2.7°C ± 0.76°C different to environmental conditions; mean ± SE absolute difference in temperature), whichever was closest to the environmental water temperature when sampled. Passion Puddle experiments had 14:10 hour light:dark cycle regimes and a light intensity of ~150 μmol quanta m^−2^ s^−1^ during the light period.

North Atlantic Ocean samples were collected during the North Atlantic Aerosol and Marine Ecosystem Study campaign (NAAMES; [47, 48]; **S1 Table in S1 File**; Experiments F-R) between March 25^th^ and April 6^th^, 2018. Samples were collected pre-dawn from 5 m depth *via* the *R/V Atlantis* flow-through seawater system (Experiments F-I and O-R), from depth using 20 L Niskin bottles (Experiments J-M; General Oceanics, USA). For Experiment N, surface microlayer samples were collected using a Garrett screen at the same site that sub-surface samples were collected for Experiment O. No permits were required for this work because sites were either locations that do not require permits to sample (Passion Puddle on the Rutgers University New Brunswick campus) or were in international waters (marine sites).

### Microtiter 96-well plate setup

100 mL of each sample were pre-filtered with sterile 40 μm mesh cup strainers to remove large particulates and then split into two sterile 50 mL centrifuge tubes (**S6 Fig in S1 File**). Nutrients (to *f*/2 rich media final nutrient concentrations; [67]) were added to one subsample (‘nutrients added’); the other subsample was unaugmented (‘no nutrients added’). For both treatments, 15 mL of sample was passed through a sterile 0.45 μm pore-size syringe-tip filter to a sterile 15 mL centrifuge tube, yielding 0.45 μm-filtered diluent free of phytoplankton and grazers. This process was repeated with a 0.02 μm syringe-tip Anotop filter for Passion Puddle experiments and with a 30 kDa nominal molecular weight cut off (NMWCO) tangential flow filter for NAAMES experiments to generate a diluent free of phytoplankton, grazers, and viruses. ‘Nutrient added’ and ‘no nutrient added’ whole waters were then aliquoted separately into acid-washed triple-rinsed multichannel reservoirs. Diluents were added to make 3 mL serial dilution series of 100%, 50%, 25%, and 12.5% whole water (1:0, 1:1, 1:2, 1:4 sample water:diluent ratios) for each nutrient treatment and diluent type (**S6 Fig in S1 File**) with a large-bore 5 mL pipette to minimize shearing. Replicate 200 μL subsamples were then aliquoted into two duplicate 96-well plates that were sampled immediately (*t* = 0 hr) or after incubation (*t = t*_*inc*_ = 24 hr; **S7 and S8 Figs in S1 File**). Because of pre-filtration for flow cytometry and small incubation volumes, the assay focused on predation by small (*i*.*e*., < 40 μm diameter), abundant (*i*.*e*., > 10^2^ cells per mL) viral and heterotrophic nanoflagellate predators.

### Phytoplankton densities quantified with flow cytometry

Cells were enumerated on a BD Accuri C6 flow cytometer, providing a census of phytoplankton cells in 35 μL—142 μL of sample (see **S9 Fig in S1 File** for initial and final counts and coefficients of variation for initial counts in all experiments and treatments). Counts were triggered by chlorophyll fluorescence (FL3-A values ≥ 1000) and plotted versus forward scatter (FSC-A), with both axes log-transformed and gates with lower thresholds set using 0.02 μm-filtered diluents. Wash cycles were run between each well. Phytoplankton concentrations were calculated by dividing counts by the volume of sample water analyzed.

### Calculation of apparent growth rates

We calculated daily apparent growth rates (AGRs) as AGR = *ln* (final phytoplankton density/initial phytoplankton density) / time in days (see Eq(1) below) across a range of dilutions [23] carried out using either 0.02 μm- or 0.45 μm-filtered diluents for total predation and grazing effects, respectively [26]. Because the initial *t = 0* and final *t = t*_*inc*_ plates were consumed by sampling (i.e., destructive sampling), we paired the *t = 0* and *t = t*_*inc*_ readings from initially identical, matching wells on these plates to calculate AGR (**S8 Fig in S1 File**).

### Mapping of experimental sites and chlorophyll

North Atlantic experimental site locations were recorded using the *R/V Atlantis* Global Positioning System (GPS) and mapped using the m_map package for Matlab (**Figs 2B and 8B**). Surface chlorophyll concentrations (**Fig 8B**) represent a monthly composite of Chlorophyll *a* for March 2018 (the NAAMES sampling period) and were provided by the NASA Goddard Space Flight Center, Ocean Ecology Laboratory, Ocean Biology Processing Group (https://earthdata.nasa.gov/) and were mapped using m_maps [68].

### Culture experiments in 96-well plates

*Emiliania huxleyi* CCMP374 was grown in either 0.2 μm-filtered autoclaved, aged seawater collected from coastal New Jersey, USA or organic matter-free and toxin-free Artificial Seawater defined media (ASW). Both types of media were amended with *f*/2 nutrients ([67]; **Fig 6B–6D**). Paired *E*. *huxleyi* and EhV experiments with similar initial viruses and hosts were aliquoted into different culture vessels (*i*.*e*., 96-well plates and 40 mL culture flasks). *E*. *huxleyi* was incubated in 2 mL 96-well plates (*c*.*f*., ~ 250 μL well volumes in all other experiments) under an 18°C, 14:10 hour light:dark cycling regime with a light intensity of ~150 μmol quanta m^−2^ s^−1^ during the light period. Cultures were counted on the Accuri flow cytometer as above.

*Dunaliella tertiolecta* was cultured in 0.02 μm-filtered seawater collected on-site in the North Atlantic with or without *f*/2 nutrients added. *D*. *tertiolecta* cultures were incubated in the sample plates and conditions as parallel mixed community experiments (Experiment R; **S1 Table in S1 File; Fig 6A**).

### Physically-explicit modeling of plate wall encounters

To estimate how many phytoplankton cells would encounter a vessel wall during an experiment and how this relationship depends on container size, we relied on the definition of the diffusion coefficient (*D*, m^2^ s^-1^) as the surface of the sphere explored by a particle within a given time interval (*Δt*, set to 24 hours for our experiments). We randomly released virtual phytoplankton cells with a concentration of 10^3^ cells per mL inside a sphere of a given volume. We then computed the radius surface explored by each particle in the volume for the duration of the experiment *Δt* as:

$$r=\sqrt{D\;\Delta t\;{\left(4\pi \right)}^{-1}}$$

This radius was compared to the initial position of each particle to evaluate whether it had encountered the wall during the interval *Δt* (**Fig 4**). Diffusion coefficients *D* that we calculated following the Stokes-Einstein equation:

$$D=\frac{kT}{6\pi \psi a}$$

where *k* is the Boltzmann constant (*k =* 1.38 *10^−23^ m^2^ kg s^-2^ K^-1^), *T* is the water temperature expressed in Kelvin, *ψ* indicates the water viscosity (set here at 8.9*10^−4^ Pa s) and *a* represents the size of the particle (m). In our analysis, we tested diffusion coefficients of 1 μm-diameter phytoplankton cells (see **S1 Fig in S1 File** for the size distribution of samples). In the case of no phytoplankton motility, the diffusion coefficient *D* derived from the Stokes-Einstein equation describing Brownian motion corresponds to 0.1 μm^2^ s^−1^. We then considered the possibility of phytoplankton motility, which was included in the model as a ‘random swimming’ at speed of ~1 μm s^−1^ and resulted in an effective diffusion coefficient of ~ 10 μm^2^ s^−1^. We repeated this numerical experiment for different volumes, *V*, to calculate their corresponding AGR resulting from the mortality of phytoplankton by wall adsorption.

### Dynamical theoretical recapitulation of the dilution assay

The theoretical basis of using dilution experiments to calculate top-down-regulated mortality rates–including grazers as well as viruses–relies on the assumption of exponential growth for the cultured population [23, 26]. If *P(t)* is the density of an incubated phytoplankton population at time *t*, and *t*_*inc*_ is the incubation time, the assumption is equivalent to the following mathematical expression:

$$P(t_{inc})=P(0)e^{AGR∙t_{inc}},$$ (1)

where AGR represents the apparent (or net) growth rate of the phytoplankton population, in turn, given by:

$$AGR=\mu -m_{tot}$$ (2)

The first term, *μ*, represents the intrinsic growth rate of the population (*i*.*e*., the cellular division rate *via* nutrient uptake and photosynthesis), whereas the second term, *m*_*tot*_, represents the mortality rate due to all possible sources. Here, we focus on top-down regulation and assume that natural mortality is negligible. We further assume that the sources for top-down regulation can be grouped into predators (*m*_*Z*_) and viruses (*m*_*V*_), leading to:

$$m_{tot}=m_{Z}+m_{V}$$ (3)

Thus, measuring the initial and final (*i*.*e*., at *t*_*inc*_) population densities provides the AGR:

$$AGR=\mu -m_{Z}-m_{V}=\frac{1}{t_{inc}}ln\left[\frac{P(t_{inc})}{P(0)}\right]$$ (4)

Let us now consider a filter with a pore size able to remove predators (*e*.*g*., commonly used 0.45 μm pore-size filtration, which also removes grazers). Let us further assume that mortality due to predators is proportional to the density of predators in the sample, *Z*, that is:

$$m_{Z}=\alpha_{Z}Z$$ (5)

where the proportionality constant, *α*_*Z*_, represents the rate of contact between predators and phytoplankton that result in death of the phytoplankton. A dilution factor *d* entails that a fraction *d* of the total sample corresponds to non-filtered water (*i*.*e*., only a fraction *d* of the total predator population remains in the otherwise untouched sample). Note that this dilution factor is opposite to the percent diluent used in our figures (% diluent = 100·(1-d)).

Including the effects of dilution leads to the expression:

$${AGR}_{0.45}=\frac{1}{t_{inc}}ln\left[\frac{P(t_{inc})}{P(0)}\right]=(\mu -m_{V})-dm_{Z}$$ (6)

Assuming that growth and mortality rates do not change with time, Eq (6) indicates that we can obtain the mortality rate due to predators as the slope resulting from a linear regression of the AGRs obtained for different dilution factors.

We can further use a filter with a pore size such that viruses are also removed (*e*.*g*., 0.02 μm pore-size Anotop filter or a 30 kDa NMWCO tangential flow filter). Thus, assuming that the mortality rate due to viruses is proportional to the viral density, *V*, that is:

$$m_{V}=\alpha_{V}V,$$ (7)

where the proportionality constant, *α*_*V*_, is the rate of encounters between viruses and phytoplankton, then the associated AGR of the sample is:

$${AGR}_{0.02}=\frac{1}{t_{inc}}ln\left[\frac{P(t_{inc})}{P(0)}\right]=\mu -dm_{V}-dm_{Z}$$ (8)

Assuming again that growth and mortality rates do not change with time, the slope of a linear regression of the apparent growth rate versus the dilution factor produces the total mortality rate, *m*_*tot*_. Using Eq (3) with the slope deduced in Eq (6) finally provides the value of the mortality rate due to viruses. Alternatively, *m*_*V*_ can be obtained by measuring the slope (and/or vertical intercept) of the curve that results from subtracting the AGRs obtained with the second and the first filters:

$${AGR}_{0.02}-{AGR}_{0.45}=\mu -dm_{V}-dm_{Z}-[(\mu -m_{V})-dm_{Z}]=m_{V}-dm_{V}$$ (9)

A compilation of the assumptions that underlie these classical dilution experiments can be found in **Table 1**.

Focusing on viruses, the assumption that predator and, particularly, viral densities stay constant during the dilution assay (Assumption 5 in **Table 1**) can lead to inaccuracies due to rapid growth of predators even over short *t*_*inc*_. To study the effect that relaxing Assumption 5 has on the AGR curve, we made a simple model for host-virus (alternatively, phytoplankton-predator) interactions based on the extensively-used phytoplankton and virus *E*. *huxleyi*-*Coccolithovirus* (EhV) system with parameterization from [62].

If *P(t)* represents the density of hosts and *V(t)* the density of viruses, the model is given by:

$$\frac{dP}{dt}=\mu_{max}\left(1-\frac{P}{K}\right)P-\alpha_{V}PV$$ (10)

$$\frac{dV}{dt}=B\alpha_{V}PV-\delta V$$ (11)

The first term in Eq (10) represents phytoplankton growth, determined by a maximum growth rate, μ_*max*_, and carrying capacity *K*. The latter term in Eq (10) accounts for growth limitation due to nutrient competition. The second term, which follows Assumption 3 (**Table 1**), represents the interaction with the virus, which the phytoplankton encounters at a contact rate *α*_*V*_ resulting in instantaneous cell lysis. Lysis produces *B* virions per cell (burst size), increasing the concentration of extracellular viruses (first term in Eq (11)), which can decay at a certain rate, *δ* (second term). This model is a variant of the classic Lotka-Volterra model that includes prey (phytoplankton, in this case) intra-specific competition through a logistic term. The parametrization and units for the model can be found in **S3 Table in S1 File**.

To recreate a natural sample, we used as an initial condition for our *in-silico* populations the long-term steady-state solution for Eqs (10)–(11), obtained by imposing *dP/dt = dV/dt = 0*:

$$P_{st}=\frac{\delta }{\alpha_{V}B}\;V_{st}=\frac{\mu_{max}}{\alpha_{V}}\left(1-\frac{\delta }{\alpha_{V}BK}\right)$$ (12)

Note that these expressions represent phytoplankton and viral densities in the original sample and, therefore, diluting the sample reduces these densities to a fraction *d·P*_*st*_ and *d·V*_*st*_, respectively.

With this model parameterized with *Emiliania huxleyi* and EhV values, we examined (i) the effects of altered incubation time for measured phytoplankton and viral AGRs (**Fig 5** and **S3 Fig in S1 File**), (ii) the effect of initial virus:host ratios (**S4 Fig in S1 File**), and (iii) dilution-induced lagged phytoplankton growth (**S5 Fig in S1 File**).

### Statistical analysis

Plots were made using the ggplot2 package in R (https://www.r-project.org/) using the stat_smooth(), lm(), and geom_density() functions to generate model I linear regression lines and histograms, respectively. Note that some experiments (*e*.*g*., Experiments D, K, N, P, and O without nutrients added and Experiments B, E, O, and P with nutrients added) may show non-linear effects that would be amenable to estimating *g* after [69, 70]. However, to ensure a consistent analysis across all experiments, we have retained all data points and analyzed all experiments with the same linear regression approach. All figures were compiled in Inkscape (https://inkscape.org/).

## Supporting information

S1 File Supplementary material. (DOCX)
