# Supplementary material for: Altered growth and death in dilution-based viral predation assays
Source: PLoS One. 2023 Jul 7;18(7):e0288114. doi: 10.1371/journal.pone.0288114 (PMC10328242; doi:10.1371/journal.pone.0288114)
Supplement: S1 File — (DOCX) [file pone.0288114.s001.docx]

Supplementary Materials for *‘Altered growth and death in dilution-based viral predation assays.’*

Ben Knowles, Juan A Bonachela, Nick Cieslik, Alice Della Penna, Ben Diaz, Nick Baetge, Michael J Behrenfeld, Karen Naumovitz, Emmanuel Boss, Jason R Graff, Kimberly H Halsey, Liti Haramaty, Lee Karp-Boss, and Kay D Bidle

**Theoretical modeling of dilution experiment dynamics**

*Limitations to the theory underlying the classic dilution experiments*

The main assumption underlying classic dilution experiments is that Eq.(6), (8) and/or (9) can be used to calculate mortality rates due to predators and/or viruses. Importantly, those expressions require that the phytoplankton population grows exponentially (Assumption 1 in **Table 1**) at a fixed growth rate (Assumption 4). The independent sources of top-down regulation should remain constant over time (Assumption 5), and be proportional to the density of the source of regulation (Assumption 3). It also requires for natural mortality to be negligible with respect to the top-down sources of mortality, or lower than the growth rate (Assumption 2).

A few of these assumptions can be defended as applicable to the typical sample and conditions under which the experiments are conducted. For example, the typical incubation time for classic dilution experiments is 24 hours, a period in which the typical sample does not draw down resources enough for death by starvation to be considered significant (Assumption 2). For the same reason (*i.e.*, the population does not have the time to reach carrying capacity), it is not an unreasonable approximation to consider that the population growth rate remains close to its maximum during the incubation period (Assumption 4). See Beckett and Weitz (2017) for theoretical results that take into account nutrient limitation.

The density-dependence of the top-down regulation rates (Assumption 3) is a standard approximation that, although overly simplistic, may apply under the well-mixed incubation environments and the typical short digestion and infection times of predators and viruses, respectively. Although Assumption 5 is not valid under any realistic scenario because both predators and viruses grow in timescales comparable to or faster than phytoplankton, conditions under which the maximum growth rate remains considerably higher than any source of mortality render Assumptions 3 and 5 useless and ensure that the phytoplankton population grows exponentially fast in the duration of the experiment (*i.e.*, Assumption 1 is valid). Initially-low predator:phytoplankton and virus:phytoplankton ratios could also result in this scenario. Under such conditions, however, the expectation is that *m_tot_ <<* *μ* and therefore the apparent growth rates obtained under different dilution factors trivially produce a flat curve, at least for the typical duration of dilution experiments (24 hours).

Nonetheless, even in such conditions, waiting a sufficiently long period of time should result in the growth of the predator and/or viral populations enough for them to influence the growth of the phytoplankton population. As explained in further detail below, the relative time between the growth of the predator/viral population and the duration of the experiment is indeed key for the ability of the theory to account for mortality rates. Thus, understanding the measured apparent growth rates requires characterizing the dynamics of the top-down regulation involved.

*The apparent growth rate curve when top-down regulation is dynamic*

In order to study the effect that relaxing (the unrealistic) assumption 5 has on the apparent growth rate curve, we used a simple model for host-virus (alternatively, phytoplankton-zooplankton) interactions. We focused on the *Emiliania huxleyi*-EhV system, for which we have our empirical collection of data that can be used to inform the model.

In this model, we focus on top-down regulation and thus we simplify the rest of the elements that are included. Following assumption 3 above, we assume that such interaction term is proportional to both the density of hosts, *P(t)*, and the density of viruses, *V(t)*. Specifically, the model is given by Eqs.(10) and (11) in the main text.

The first term in Eq.(10) represents the growth of the host feeding on a specific resource(s), with a maximum growth rate *μ_max_* and a carrying capacity set by *K*. Including the latter allows us to represent a potential nutrient limitation due to competition for the available resource in a simple way (Beckett and Weitz 2017); if nutrients are not limiting, the term describes an approximate exponential growth. The second term represents the interaction with the virus, which the host encounters at a contact rate *α_V_*, and which results in (instantaneous) cell lysis. Lysis produces *B* virions per cell, which provides the new extracellular viruses (the first term in Eq.(11)), which can decay at a certain rate, *δ* (second term). This simple model is a variant of the classic Lotka-Volterra model that includes prey (host, in this case) intraspecific competition through a logistic term. The parametrization and units for the model can be found in **Supplementary Table 3**.

In order to recreate a natural sample from a system represented by this model, we used as initial condition of the undiluted sample the long-term stationary state for these equations, obtained by imposing *dP/dt = dV/dt = 0* (see Eqs.(12) and (13) in main text). The initial host and viral density in the original diluted sample, *P_0_* and *V_0_*, will thus be a fraction *d* of these densities:

| $P(0)=d\cdot P_{st}$; $V(0)=d\cdot x_{0}\cdot P_{st}$ | (S1) |
| --- | --- |

The factor *x_0_* allows us to explore the effect of imposing a specific initial virus-to-host ratio, with *x_0_* ranging from 0.1 to 300. Setting $x_{0}=V_{st}/P_{st}$recovers the virus:host ratio from the original sample (*i.e.*, Eq.(13) divided by Eq.(12)).

The initial ratio virus-to-host, together with the incubation time, proved to be the features of the simulated experimental setup that influenced the most the emergent apparent growth rate curves.

*The importance of incubation time*

When simulating numerically the system provided by Eqs.(10)-(13) and (S1) (using the parametrization from **Supplementary Table 3**) our results indicated that, given a *long enough* incubation time, the host population shows an initial exponential growth that eventually turns into population decline. **Supplementary Figure 3** (upper-left panel) shows such behavior for several dilution factors, for an incubation time of *t_inc_*=9 d.

The different stages in population growth within a dilution factor, and the relative performance across dilution factors, define different behaviors for the apparent growth rate curves. Consequently, the resulting apparent growth rate (AGR) curve would show very different slopes depending on the incubation time chosen for dilution experiments (**Supplementary Figure 3,** upper-right panel). For the typical incubation time (*t_inc_*=1 d, red), the population is still growing exponentially fast as the viral population has not grown yet to high levels (**Supplementary Figure 3** lower-left panel shows that in such time the most viral density has grown in that time is 50%). For that incubation time, the associated host AGR shows a constant positive slope (*i.e.*, a linear behavior). If the incubation time were set to 3 or 4 days instead (green and blue), the experiment would be stopped when the host population starts slowing down due to the (now noticeable) viral pressure for all percent diluents but, due to the density dependent relationship that defines the host-virus interaction, the higher percent diluents show a faster decline. As a consequence, the resulting AGR shows a milder slope for initial and intermediate percent diluent than it does for higher values. Increasing the incubation time to 6 days (orange, **Supplementary Figure 3** upper panels) implies that the population density is measured when the population is declining, although it still remains above its initial value for all dilution factors (and it is barely declining for lower percent diluents). This leads to an AGR curve that shows an almost negligible slope at first, but keeps increasing as the percent diluent increases. For an incubation time of 8 days (purple curve, **Supplementary Figure 3** upper panels), all dilutions show a population density below its initial value, with the distance to such value decreasing as the percent diluent decreases. As a consequence, the associated AGR shows a negative slope that converges to zero as the percent diluent increases.

To understand better the resulting AGR curves for the host, we also measured the apparent growth rate for the viral population, given by:

| ${AGR}_{V}=\frac{1}{t_{inc}}ln\left[ \frac{V\left( t_{inc} \right)}{V(0)} \right]$ | (S2) |
| --- | --- |

As the lower-right panel of **Supplementary Figure 3** shows, the viral AGR (AGR_V_) curve shows a negative slope for incubation times for which the host population is still in the exponential growth stage (red curve). If the incubation time is such that the host departs from such regime for some dilution factors, the AGR_V_ curve shows a non-monotonic behavior in which the slope transitions to positive for those factors (green and blue curves, low percent diluent; **Supplementary Figure 3**, lower panels). For incubation times for which the host population is declining for any percent diluent, the AGR_V_ curve shows a positive slope. The closer the host is to exponential growth for all *d* values, the closer the AGR_V_ curve is to linearity.

Importantly, the model represented by Eqs.(10)-(11) predicts that host and virus will eventually reach a stationary state that does not depend on the initial conditions. In other words, for a sufficiently long incubation time, the host-virus dynamics will break Assumption 1. Interestingly, this stationary state can be reached monotonically or *via* damped oscillations. Either of these scenarios could be described by **Figure 5** and **Supplementary Figure 3**, *i.e.*, AGR and AGR_V_ curves with a slope that changes as incubation time increases.

In summary, dilution experiments require measuring population densities when the host-virus system is still showing transient behavior. Thus, the resulting AGR (both slope and shape) and AGR_V_ curves are very sensitive to the incubation time, mostly because the viral density does not remain constant over time but instead grows and eventually subdues the host population.

*The importance of the initial ratio virus:host*

The point where the viral pressure forces the decline of the host population changes with the initial virus-to-host ratio (*x_0_*). High virus-to-host ratios lead to situations like the one depicted in **Supplementary Figure 4** (*x_0_* ≈ 300), in which the effects of the viral population (and the absence of it via dilution) are noticed early in the incubation. As the ratio decreases, the initial viral pressure on the host decreases and the differences across dilution factors become less noticeable (results not shown). For identical incubation times (*t_inc_* = 1 d) a lower initial ratio increases the absolute value of the slope as all dilution factors show a behavior similar to the high percent diluent cases, in which the virus is scarce. The shape of the curve, however, changes, as incubation time increases, and depending on the incubation time slopes that could be either positive or negative are possible.

*Linear AGR curves can be obtained via growth lag even in the absence of mortality*

Our dilution experiments in the absence of predators/viruses show a negative “mortality” rate (*i.e.*, a negative AGR slope), which leads to the hypothesis that dilution-related physiological effects on the host population may result in initial lags that ultimately translate into such an AGR curve (**Supplementary Figure 4**).

To test this hypothesis, we devised a virus-free version of our model (*i.e.*, only Eq.(10) with $\alpha_{V}=0$) in which we added an effect of dilution on the population growth rate:

| $\frac{dP}{dt}=\mu(t)\left( 1-\frac{P}{K} \right)P$ | (S3) |
| --- | --- |

where:

| $\mu(t)=\left\{ \begin{matrix} \mu_{max}\frac{t}{t_{lag}} \\ \\ \mu_{max} \end{matrix}\begin{matrix} if t<t_{lag} \\ \begin{matrix} \\ \end{matrix} \\ if t\geq t_{lag} \end{matrix} \right.$ | (S4) |
| --- | --- |

In other words, we assumed that dilution affects the physiology of the host population, which needs a certain time to recover. We further assumed that such a time increases with dilution (*i.e.*, decreases with the proportion of unfiltered water, *d*) up to a maximum; specifically:

| $t_{lag}=t_{{lag}_{MAX}}\left( 1-d \right)$ | (S5) |
| --- | --- |

Thus, total dilution leads to the maximum lag ($t_{{lag}_{MAX}}$), and no dilution translates into no lag (**Supplementary Figure 5**). Note that we assumed the simplest possible (*i.e.*, linear) forms for Eq.(S4) and (S5), but the expectation is that our conclusions hold as long as the lag correlate positively with dilution.

Our results show AGR curves obtained for several values of the maximum lag, $t_{{lag}_{MAX}}$. For a given incubation time, the longer the maximum lag the more negative the slope of the resulting AGR is (*i.e.*, the more negative the “mortality” rate that would be measured from it; **Supplementary Figure 5**). Because there is no mortality (and no nutrient limitation for usual incubation times), all populations grow monotonically and therefore incubation time does not alter the qualitative pattern. Incubation time, however, does decrease the steepness of the AGR curves, since the differences across dilutions become less apparent as incubation times are chosen larger and larger than the maximum lag.

*AGR curves still provide valuable information on mortality…we just don’t know how to extract rates*

The theory underlying dilution experiments is constrained by the set of assumptions compiled in **Table 1** (main text). Although these assumptions enable an analytical derivation of the expressions that indicate how dilution can potentially be used to deduce mortality rates, these assumptions are not applicable under most realistic situations. Moreover, our results show that, because the sample is disturbed by the dilution process, incubation can lead to very dramatic changes in the density of both host and virus. Therefore, host mortality changes with time (*i.e.*, there is no single number that can represent the mortality present in the original sample). Despite this failure to quantify mortality rates, there is a wealth of valuable information that can help understand how top-down sources contribute to the focal phytoplankton population.

Our theoretical results can help improve the experimental protocols to ensure an accurate description of such top-down regulation. The results above describe a quite heterogeneous landscape of possible AGR curves that can be obtained in a dilution experiment. The shape and slope of the AGR curves depend on key factors such as initial growth lags, the ratio of extracellular viruses-to-host-to-intracellular viruses, and incubation time. All these factors can ultimately be summarized in the relative growth of the host population and the source(s) of top-down regulation, which should be taken into consideration when deciding on the incubation time (**Figure 5, 7, and 8**).

For example, incubation time needs to be sufficiently long for the virus/zooplankton populations to grow to densities that significantly influence the phytoplankton population. However, incubation should not be as long as to allow for top-down regulation to trigger a decline in the host population, nor for nutrients to limit phytoplankton growth. Experimental protocols already address the latter by conducting dilution experiments under nutrient-rich conditions, but enriching the original sample may lead to a departure of the typical nutrient conditions for the sample, which in turn may result in altered host physiology (and therefore in viral production; Choua and Bonachela 2019).

In addition, we show that measuring viral population densities at the beginning and the end of incubation helps build expectations regarding (and in the interpretation of) the AGR curve. Such additional measurements provide the initial virus:host ratio, which our results show influences deeply the slope of the AGR curve. Moreover, these measurements enable the calculation of a viral apparent growth rate (AGR_V_) curve, which summarizes the behavior of the viral population and, together with the host’s AGR, provides a more complete picture of their dynamics. For example, a flat AGR_V_ curve would confirm the validity of assumption 4 (grazer/viral population did not change with time for any of the dilution factors). If the curve is flat but not zero, it indicates that the population has changed proportionally to the change in the dilution factor, *i.e.*, keeping a similar (but nonzero) distance between final and initial densities. A negative slope indicates that the difference between the viral density at the end and the beginning of the incubation is less negative (or more positive) for lower percent diluents, *i.e.*, the viral population performs better as the percent diluent decreases. The typical consequence is that the host population shows a larger decimation as the dilution factor decreases, *i.e.*, larger mortality (or, if viral pressure is not high enough, negligible mortality). A positive slope in the AGR_V_ curve indicates that the difference between final and initial densities for the virus is less positive (or more negative) as the percent diluent decreases; in other words, high percent diluents result in a better performance of the virus, and it is for those *d* values that the host experiences higher mortality.

**Figure 5** shows that providing additional information on whether the AGR_V_ curve increases or decreases for a specific range of dilution factors enables a more complete picture of the mortality in the original sample. Increasing mortality with the percent diluent (positive slope for the AGR_V_ curve) is a preliminary indication that the viral population is declining at the moment of the snapshot which, with our simple model, happens because the host population has been decimated enough to decline (positive AGR slope) or decimation is starting to slow down (*i.e.*, population recovering after declining, negative AGR slope). Decreasing mortality with the percent diluent (negative slope for the AGR_V_ curve) should result from a growing host population; if host density is starting to decelerate markedly or even starting its decline, the AGR_V_ curve transitions from negative to positive slope (**Figure 5**).

Moreover, the slope of AGR curve by itself cannot be used to understand the dynamics of the host (the AGR_V_ curve seems to be a better predictor). Assuming that top-down regulation is present, a growing host population could result in a negative or positive AGR slope depending on whether the viral population is growing (red curves in **Supplementary Figure 3**) or declining (purple curves), respectively (also see **Figure 5**). On the other hand, the classical expectation (positive and approximately constant slope for the AGR curve) seems to result from only one possible scenario under reasonable conditions: host (still) growing exponentially fast, and virus at density levels that are not high enough to slow down markedly the host population.

**References**

Beckett, S. & Weitz, J. (2017). Disentangling niche competition from grazing mortality in phytoplankton dilution experiments. *PLoS One*, *12*(5), e0177517.

Choua M. & Bonachela, J.A. (2018). Ecological and evolutionary consequences of viral plasticity. *The American Naturalist*, *193*(3), 346-358.

Knowles, B., Bonachela, J.A., Behrenfeld, M.J., Bondoc, K..G, Cael, B.B., Carlson, C.A., Cieslik, N, Diaz, B.P., Fuchs, H.L., Graff, J..R, Grasis, J., Halsey, H., Haramaty, L., Johns, C.T., Natale, F., Nissimov, J.I., Schieler, B., Thamatrakoln, K., Thingstad, T.F., Våge, S., Watkins, C., Westberry, T., & Bidle, K.D. (2020). Temperate infection in virus-host model system previously known for virulent dynamics. *Nature Communications.* doi: 10.1038/s41467-020-18078-4

**Supplementary Table and Figure Legends**

**Supplementary Table 1: Summary of experimental dates, sites, and conditions (mapped in Figures 1b and 7b).** See Behrenfeld et al (2019) for further information on NAAMES stations. Note that Experiments N and O were conducted with water collected from the same site, using 5 m subsurface water and surface microlayer water, respectively. Note that incubation temperatures were similar to environmental conditions in all experiments: incubation temperatures were within 2.7 °C ± 0.76 °C (mean absolute difference ± SE between column 8 and 9 values) in Experiments A-E and the same as the surface ocean in Experiments F-R.

**Supplementary Table 2: Linear regression summary statistics for apparent growth rates (AGRs) across dilution levels, with and without nutrients added (Figure 2b).** All numbers rounded to two significant figures; 0.00 values were < 0.005.

**Supplementary Table 3: Symbols, units, and parametrization used for the different density-dependent models.** Note that the model is parameterized using the well-studied *Emiliania huxleyi*-EhV *Coccolithovirus* system as a reference.

**Supplementary Figure 1: Size structure of phytoplankton communities during experiments.** Cell densities in each 1 μm-wide size bin in undiluted and highly diluted (100 % and 12.5 % seawater, respectively) treatments at the start and end of the experiments (initial and final values, respectively). Panel (**a**) shows the change in cell densities in each size bin over the experiments without nutrients added with initial and final samples shown as black and blue bars, respectively. Panel (b) shows the change in cells densities in each size fraction where nutrients were added with initial and final samples shown as red and purple bars, respectively. The location of each experiment is indicated as in **Figure 1** by horizontal color bars above each row of plots (pink = Passion Puddle in New Jersey, orange = Sargasso Sea/Gulf Stream, green = sub-tropical North Atlantic, and purple = temperate North Atlantic sites). Note that cell densities are shown as both natural and base-10 transformed (*ln* and log_10_; values on the *ln* axes are grey and italicized for clarity) for comparison with other figures in the paper.

**Supplementary Figure 2: Chlorophyll:cell size of phytoplankton at experimental sites estimated by flow cytometry.** Cellular chlorophyll quotas, measured as the chlorophyll fluorescence of each cell divided by its size forward scatter per cell) and then averaged from each sample. Note that data are color-coded by nutrient addition and experiments are color-coded in by site water mass and divided by vertical lines (see also **Figure 1**). No data exists for the nutrient unamended treatment of Experiment F, marked by the # symbol.

**Supplementary Figure 3: The importance of time in estimating predation from a change in phytoplankton and viral densities.** Top left: Dynamic behavior of the host population for several dilution factors when the system is initialized using Eqs.(12)-(14) and the original virus-to-host ratio, $x_{0}=V_{st}/H_{st}$. The color bars indicate specific snapshots of the system, with which the curves on the top right panel are obtained. Top right: Apparent growth rate defined as in Eq.(4) for the snapshots color-coded on the left panel. Bottom left: Dynamics for viral densities. Bottom right: apparent growth rate for the virus, *i.e.*, $\lambda_{V}=\frac{1}{t_{inc}}ln\left[ \frac{V\left( t_{inc} \right)}{V(0)} \right]$.

**Supplementary Figure 4: The effect of initial virus:host ratio on experimental apparent growth rates (AGR) of hosts (left) and viruses (right) under dilution.**

**Supplementary Figure 5: The effect of dilution-induced lagged growth on experimental apparent growth rates (AGR).** AGR curves were obtained with the model in the absence of top-down regulation when including explicitly the disruptive effect of dilution on host population physiology. Left: for an incubation time *t_inc_* = 1 d. Right: for an incubation time *t_inc_* = 2 d.

**Supplementary Figure 6: Flow chart of experimental setup.** Site water was passed through a 40 μm mesh to remove particulates. Nutrients were then added to one subsample of this water (upper black oval) and not to the other (upper red oval). These subsamples were either left unfiltered (grey or pink ovals) or filtered with 0.02 μm (lower black or red ovals). Each of these filtrates was then mixed with the unfiltered water in increasing ratios of unfiltered:filtered diluent (decreasing grey or pink rectangles and increasing diluent rectangles of black or red).

**Supplementary Figure 7: Plate layout for different experiments.** Experiments spanned (**a**) Passion Puddle Experiments A-E, (**b**) North Atlantic Ocean Experiments F-O, and (**c**) North Atlantic Ocean Experiments P-R. Wells are color-coded by whether nutrients were added (red circles and fractions) or not (black circles and fractions). Grey wells represent discontinued treatments. Fractions (1, 1/2, 1/4, 1/8) inside wells represent the fraction of undiluted water in each well, with undiluted, 50 %, 25 %, and 12.5 % undiluted treatments in all experiments, respectively. Note that the plate layouts provided n = 4 replicates for each treatment (dilution level, nutrient addition, diluent type) for experiments A – E, n = 3 for experiments F – O, and n = 6 for experiments P and Q.

**Supplementary Figure 8: Schematic of how rates were calculated from paired wells between the two plates.** The figure shows how the growth rate for the same in well A1 was calculated by pairing phytoplankton densities measured in well A1 at _t0_ and _t24_. The same was applied to all subsequent wells; well B1_t0_ values were paired with B1_t24_ values, C1_t0_ with C1_t24_, *etc.*

**Supplementary Figure 9:** **Cells counted in all experiments, dilution levels, and time points with associated coefficients of variation for initial counts.** Cells counted at the start of experiments (*t*_0_; *x*-axis; log_10_-transformed) are plotted against the relevant final counts (*t*_24_; *y*-axis; log_10_-transformed) across all dilution levels (87.5 % diluent, 75 % diluent, 50 % diluent, and undiluted 0 % diluent) for all experiments (columns; each experiment indicated by the letters in the grey boxes at the top of each column). Data points are color-coded by whether nutrients were added or not; nutrient unamended data are shown as black data points in the left panel and nutrient-added data as red data points in the right panel. Coefficients of variation for *t*_0_ values are shown in each plot. Note that coefficients of variation are the variance as a percentage of the mean for a given data set, calculated without log-transforming the data.

**Supplementary Tables and Figures**

**Supplementary Table 1: Summary of experimental dates, sites, and conditions (mapped in Figures 1b and 7b).** See Behrenfeld et al (2019) for further information on NAAMES stations. Note that Experiments N and O were conducted with water collected from the same site, using 5 m subsurface water and surface microlayer water, respectively. Note that incubation temperatures were similar to environmental conditions in all experiments: incubation temperatures were within 2.7 °C ± 0.76 °C (mean absolute difference ± SE between column 8 and 9 values) in Experiments A-E and the same as the surface ocean in Experiments F-R.

| *Date* | *Experiment* | *Location* | *Sampled* | *NAAMES Station* | *Latitude* | *Longitude* | *Water (°C)* | *Incubation (°C)* |
| --- | --- | --- | --- | --- | --- | --- | --- | --- |
| 2/8/2018 | A | Passion Puddle | Bottle | - | 40.48194 | 74.43722 | 3.5 | 4 |
| 2/15/2018 | B | Passion Puddle | Bottle | - | 40.48194 | 74.43722 | 7 | 4 |
| 2/22/2018 | C | Passion Puddle | Bottle | - | 40.48194 | 74.43722 | 11.5 | 15 |
| 3/01/2018 | D | Passion Puddle | Bottle | - | 40.48194 | 74.43722 | 10.5 | 15 |
| 3/8/2018 | E | Passion Puddle | Bottle | - | 40.48194 | 74.43722 | 6 | 4 |
| 3/24/2018 | F | North Atlantic | Flow-through | - | 31.34358 | 53.18482 | 21.1 | 21.1 |
| 3/25/2018 | G | North Atlantic | Flow-through | - | 34.7503 | 48.99817 | 20.1 | 20.1 |
| 3/26/2018 | H | North Atlantic | Flow-through | - | 37.5388 | 45.56232 | 19.6 | 19.6 |
| 3/27/2018 | I | North Atlantic | Niskin | 1 | 39.4097 | 43.42683 | 18.8 | 18.8 |
| 3/28/2018 | J | North Atlantic | Niskin | 2 | 39.27802 | 41.21133 | 17.6 | 17.6 |
| 3/30/2018 | K | North Atlantic | Niskin | 3 | 43.5167 | 42.21347 | 18.6 | 18.6 |
| 4/1/2018 | L | North Atlantic | Niskin | 4 | 44.47603 | 38.27997 | 13.7 | 13.7 |
| 4/3/2018 | M | North Atlantic | Niskin | 2RD | 39.875 | 39.83135 | 17.8 | 17.8 |
| 4/5/2018 | N | North Atlantic | Garett screen | µ-layer; 2RF | 39.173 | 40.12453 | 18.1 | 18.1 |
| 4/5/2018 | O | North Atlantic | Flow-through | 2RF | 39.173 | 40.12453 | 18.1 | 18.1 |
| 4/6/2018 | P | North Atlantic | Flow-through | 2RF | 39.173 | 40.12453 | 18.1 | 18.1 |
| 4/10/2018 | Q | North Atlantic | Flow-through | - | 42.08968 | 60.0747 | 14.0 | 14.0 |
| 4/11/2018 | R | North Atlantic | Flow-through | - | 40.9229 | 65.72805 | 14.4 | 14.4 |

**Supplementary Table 2: Linear regression summary statistics for apparent growth rates (AGRs) across dilution levels, with and without nutrients added (Figure 2b).** All numbers rounded to two significant figures; 0.00 values were < 0.005.

| *Experiment* | *Nutrients* | *y-intercept* | *Slope* | *Residual Std. Error* | *Adjusted r^2^* | *Degrees of Freedom* | *p-value* |
| --- | --- | --- | --- | --- | --- | --- | --- |
| A | (-) | -1.04 | -0.05 | 0.31 | -0.07 | 14 | 0.84 |
| B | (-) | -1.34 | -0.69 | 0.23 | 0.51 | 14 | 0.00 |
| C | (-) | -0.80 | -0.87 | 0.26 | 0.56 | 14 | 0.00 |
| D | (-) | -0.03 | 0.33 | 0.21 | 0.19 | 14 | 0.05 |
| E | (-) | 0.09 | 0.73 | 0.24 | 0.51 | 14 | 0.00 |
| G | (-) | -3.20 | 1.28 | 0.34 | 0.62 | 10 | 0.00 |
| H | (-) | -2.94 | 0.14 | 1.21 | -0.10 | 10 | 0.89 |
| I | (-) | -1.50 | 1.22 | 0.72 | 0.21 | 10 | 0.08 |
| J | (-) | -0.80 | 0.10 | 0.09 | 0.06 | 10 | 0.22 |
| K | (-) | -0.62 | 0.14 | 0.18 | -0.02 | 10 | 0.39 |
| L | (-) | -1.03 | -0.31 | 0.13 | 0.38 | 10 | 0.02 |
| M | (-) | -1.01 | -0.58 | 0.16 | 0.62 | 10 | 0.00 |
| N | (-) | -2.36 | 0.76 | 0.30 | 0.41 | 10 | 0.02 |
| O | (-) | -0.53 | 0.23 | 0.29 | -0.01 | 10 | 0.37 |
| P | (-) | -1.27 | 0.39 | 0.23 | 0.23 | 22 | 0.01 |
| Q | (-) | -1.74 | -0.56 | 0.42 | 0.15 | 21 | 0.04 |
| R | (-) | -1.02 | -0.30 | 0.60 | -0.02 | 18 | 0.47 |
| A | (+) | -0.99 | 0.19 | 0.45 | -0.05 | 14 | 0.58 |
| B | (+) | -0.97 | 0.01 | 0.20 | -0.07 | 14 | 0.94 |
| C | (+) | -0.38 | 0.05 | 0.23 | -0.06 | 14 | 0.76 |
| D | (+) | -0.25 | -0.05 | 0.30 | -0.07 | 14 | 0.83 |
| E | (+) | -0.57 | 0.10 | 0.35 | -0.06 | 14 | 0.70 |
| F | (+) | -2.55 | -0.84 | 0.26 | 0.51 | 7 | 0.02 |
| G | (+) | -2.76 | -0.07 | 0.41 | -0.10 | 10 | 0.84 |
| H | (+) | -1.93 | -0.30 | 0.18 | 0.21 | 10 | 0.08 |
| I | (+) | -0.94 | 0.69 | 0.40 | 0.21 | 10 | 0.07 |
| J | (+) | -0.78 | -0.02 | 0.11 | -0.10 | 10 | 0.86 |
| K | (+) | -0.91 | -0.49 | 0.15 | 0.54 | 10 | 0.00 |
| L | (+) | -0.79 | -0.08 | 0.14 | -0.05 | 10 | 0.52 |
| M | (+) | -0.98 | -0.44 | 0.18 | 0.40 | 10 | 0.02 |
| N | (+) | -1.80 | 0.03 | 0.12 | -0.09 | 10 | 0.78 |
| O | (+) | -0.35 | 0.10 | 0.15 | -0.03 | 10 | 0.44 |
| P | (+) | -1.16 | 0.07 | 0.18 | -0.03 | 22 | 0.52 |
| Q | (+) | -1.73 | -1.16 | 0.40 | 0.50 | 19 | 0.00 |
| R | (+) | -0.68 | 0.52 | 0.57 | 0.04 | 18 | 0.19 |

**Supplementary Table 3: Symbols, units, and parametrization used for the different density-dependent models.** Note that the model is parameterized using the well-studied *Emiliania huxleyi*-EhV *Coccolithovirus* system as a reference.

| *Symbol* | *Definition* | *Units* | *Value* | *Source* |
| --- | --- | --- | --- | --- |
| *t* | Time | *days* | Variable | - |
| *P* | Observed phytoplankton pop. density | *cells·L^-1^* | Variable | - |
| *V* | Concentration free viruses | *viruses·L^-1^* | Variable | - |
| *μ* | Phytoplankton growth rate | *days^-1^* | Variable | - |
| *μ_max_* | Maximum growth rate | *days^-1^* | 1.01 (Ehux in *f*/2 medium) | Knowles et al (2020) |
| *K* | Carrying capacity | *cells·L^-1^* | 6.5·10^9^ (Ehux in *f*/2 medium) | Knowles et al (2020) |
| *α_V_* | Viral adsorption rate | *L·days^-1^* | 1.44·10^-8^ | Knowles et al (2020) |
| *δ* | Viral decay rate | *days^-1^* | 1/3 | Knowles et al (2020) |
| *L* | Viral latent period | *days* | 2 | Knowles et al (2020) |
| *B* | Viral burst size | *Viruses/*  *infection* | 100 | Knowles et al (2020) |
| *t_inc_* | Incubation time | *days* | 1 - 10 | - |
| *d* | Dilution factor (fraction unfiltered water) | *-* | 0 - 1 | - |
| *λ* | Apparent growth rate (AGR) | *days^-1^* | Variable | - |
| *m_V_* | Mortality rate due to viruses | *days^-1^* | Variable | - |
| *m_G_* | Mortality rate due to grazers | *days^-1^* | Variable | - |
| *m_tot_* | Total mortality rate by top-down regulation | *days^-1^* | Variable | - |

**Supplementary Figures**

**Supplementary Figure 1: Size structure of phytoplankton communities during experiments.** Cell densities in each 1 μm-wide cell diameter size bin in undiluted and highly diluted (100 % and 12.5 % seawater, respectively) treatments at the start and end of the experiments (initial and final values, respectively). Panel (**a**) shows the change in cell densities in each size bin over the experiments without nutrients added with initial and final samples shown as black and blue bars, respectively. Panel (b) shows the change in cells densities in each size fraction where nutrients were added with initial and final samples shown as red and purple bars, respectively. The location of each experiment is indicated as in **Figure 1** by horizontal color bars above each row of plots (pink = Passion Puddle in New Jersey, orange = Sargasso Sea/Gulf Stream, green = sub-tropical North Atlantic, and purple = temperate North Atlantic sites). Note that cell densities are shown as both natural and base-10 transformed (*ln* and log_10_; values on the *ln* axes are grey and italicized for clarity) for comparison with other figures in the paper. Note that the ’10 μm’ includes cells with diameters of 10 μm and greater.

**Supplementary Figure 2: Chlorophyll:cell size of phytoplankton at experimental sites estimated by flow cytometry.** Cellular chlorophyll quotas, measured as the chlorophyll fluorescence of each cell divided by its size forward scatter per cell) and then averaged from each sample. Note that data are color-coded by nutrient addition and experiments are color-coded in by site water mass and divided by vertical lines (see also **Figure 1**). No data exists for the nutrient unamended treatment of Experiment F, marked by the # symbol.


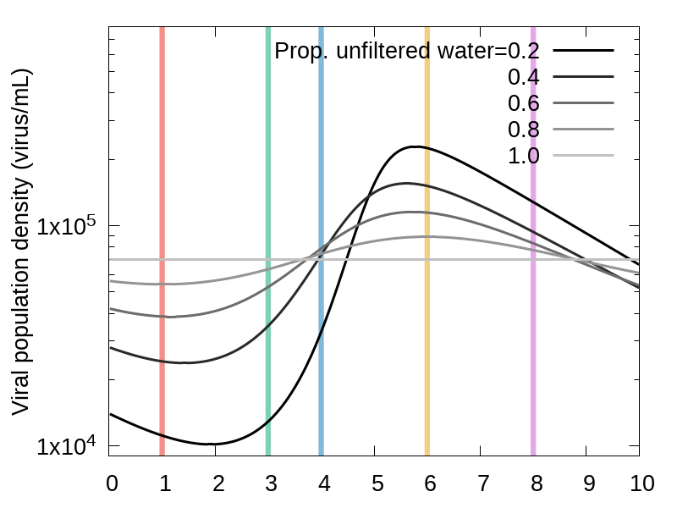
**
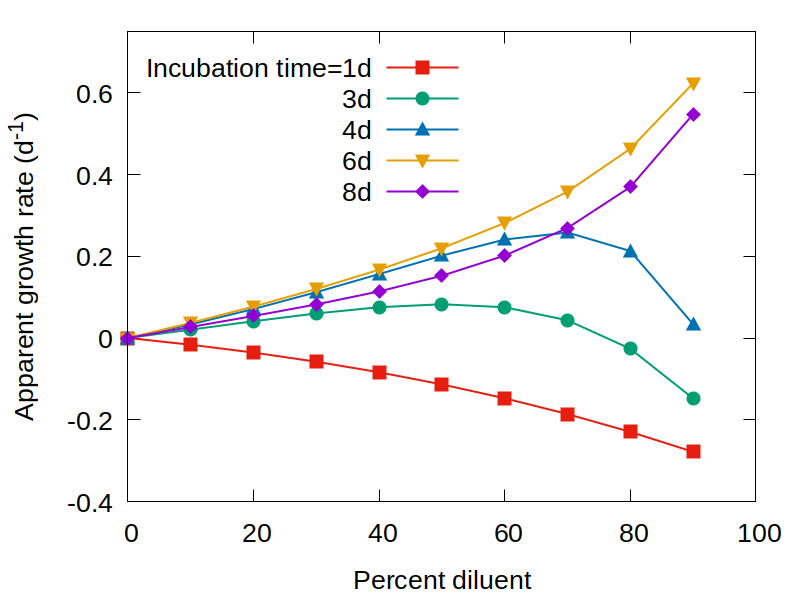
**

| 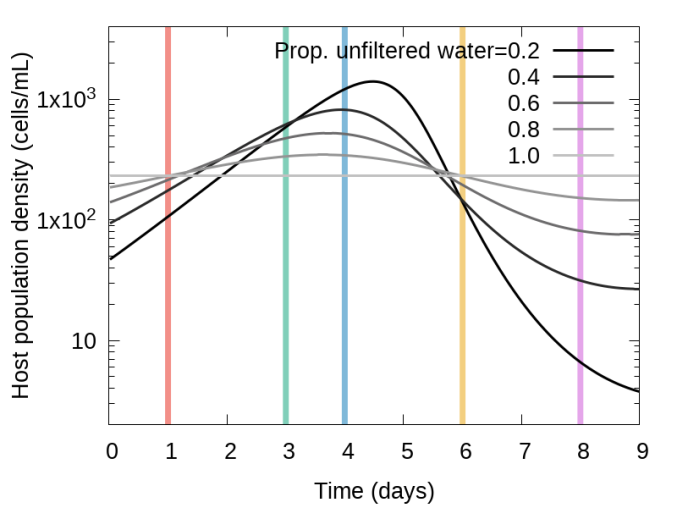 | 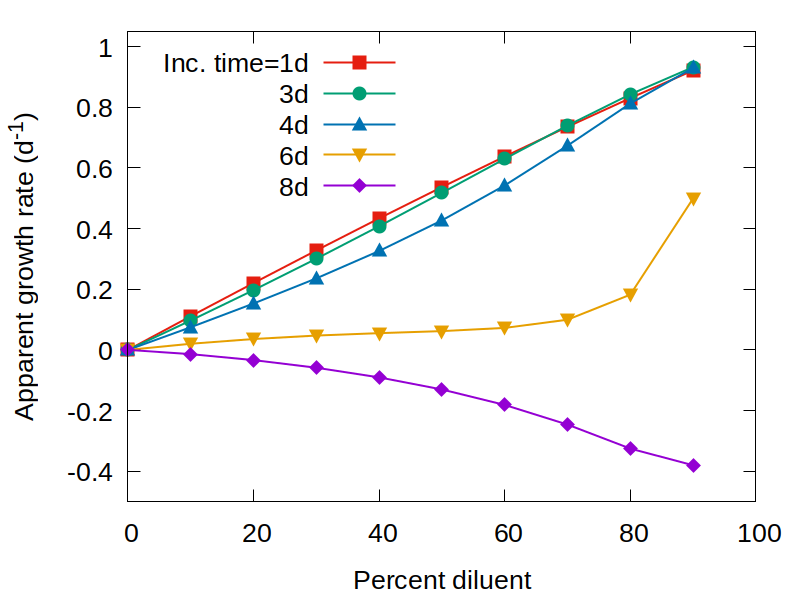 |
| --- | --- |

**Supplementary Figure 3: The importance of time in estimating predation from a change in phytoplankton and viral densities.** Top left: Dynamic behavior of the host population for several dilution factors, when the system is initialized using Eqs.(12)-(14) and the original virus-to-host ratio, $x_{0}=V_{st}/H_{st}$. The color bars indicate specific snapshots of the system, with which the curves on top right panel are obtained. Top right: Apparent growth rate defined as in Eq.(4) for the snapshots color-coded on the left panel. Bottom left: Dynamics for viral densities; the color bars represent the incubation times/snapshots from **Supplementary Figure 8**. Bottom right: apparent growth rate for the virus, *i.e.*, $\lambda_{V}=\frac{1}{t_{inc}}ln\left[ \frac{V\left( t_{inc} \right)}{V(0)} \right]$.

**
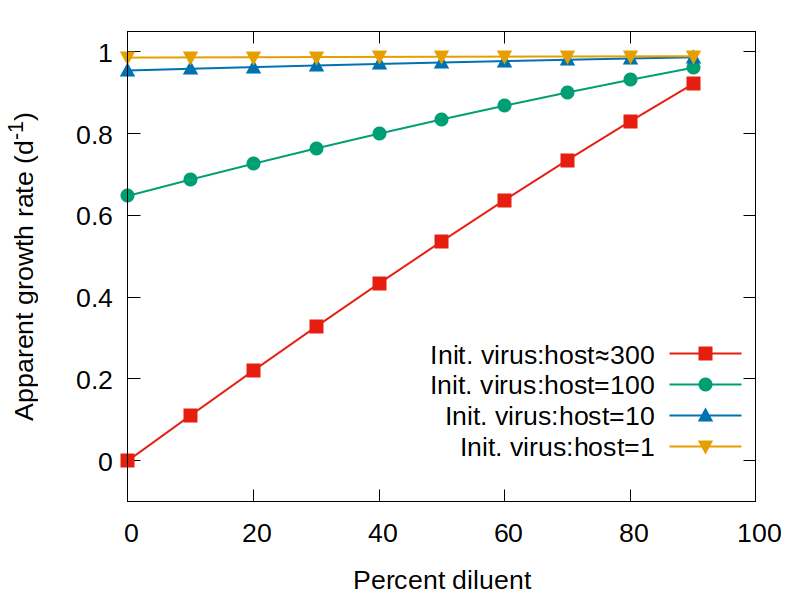

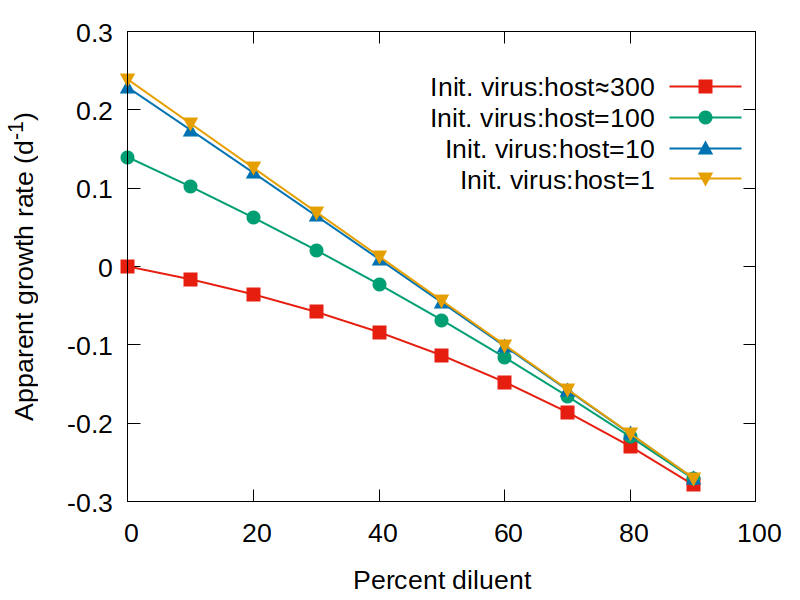
**

**Supplementary Figure 4: The effect of initial virus:host ratio on experimental apparent growth rates (AGR) of hosts (left) and viruses (right) under dilution.**

**Supplementary Figure 5: The effect of dilution-induced lagged growth on experimental apparent growth rates (AGR).** AGR curves obtained with the model in the absence of top-down regulation when including explicitly the disruptive effect of dilution on host population physiology. Left: for an incubation time *t_inc_* = 1 d. Right: for an incubation time *t_inc_* = 2 d.


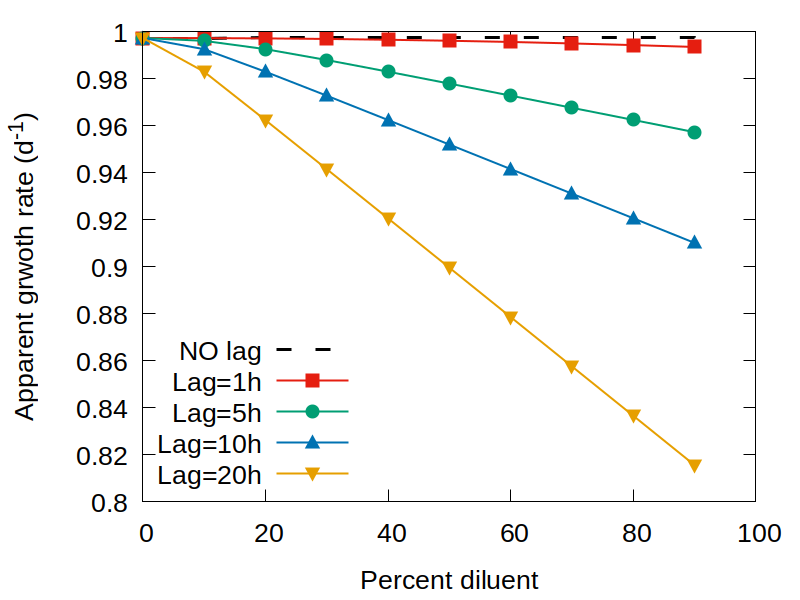

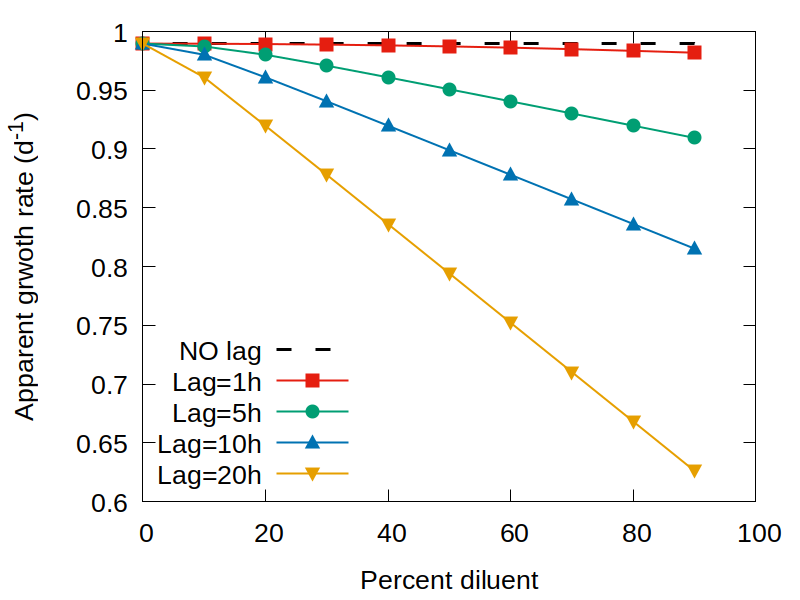


** Supplementary Figure 6: Flow chart of experimental setup.** Site water was passed through a 40 μm mesh to remove particulates. Nutrients were then added to one subsample of this water (upper black oval) and not to the other (upper red oval). These subsamples were either left unfiltered (grey or pink ovals) or filtered with 0.02 μm (lower black or red ovals). Each of these filtrates were then mixed with the unfiltered water in increasing ratios of unfiltered:filtered diluent (decreasing grey or pink rectangles and increasing diluent rectangles of black or red).

**Supplementary Figure 7: Plate layout for different experiments.** Experiments spanned (**a**) Passion Puddle Experiments A-E, (**b**) North Atlantic Ocean Experiments F-O, and (**c**) North Atlantic Ocean Experiments P-R. Wells are color-coded by whether nutrients were added (red circles and fractions) or not (black circles and fractions). Grey wells represent discontinued treatments. Fractions (1, 1/2, 1/4, 1/8) inside wells represent the fraction of undiluted water in each well, with undiluted, 50 %, 25 %, and 12.5 % undiluted treatments in all experiments, respectively. Note that the plate layouts provided n = 4 replicates for each treatment (dilution level, nutrient addition, diluent type) for experiments A – E, n = 3 for experiments F – O, and n = 6 for experiments P and Q.

**
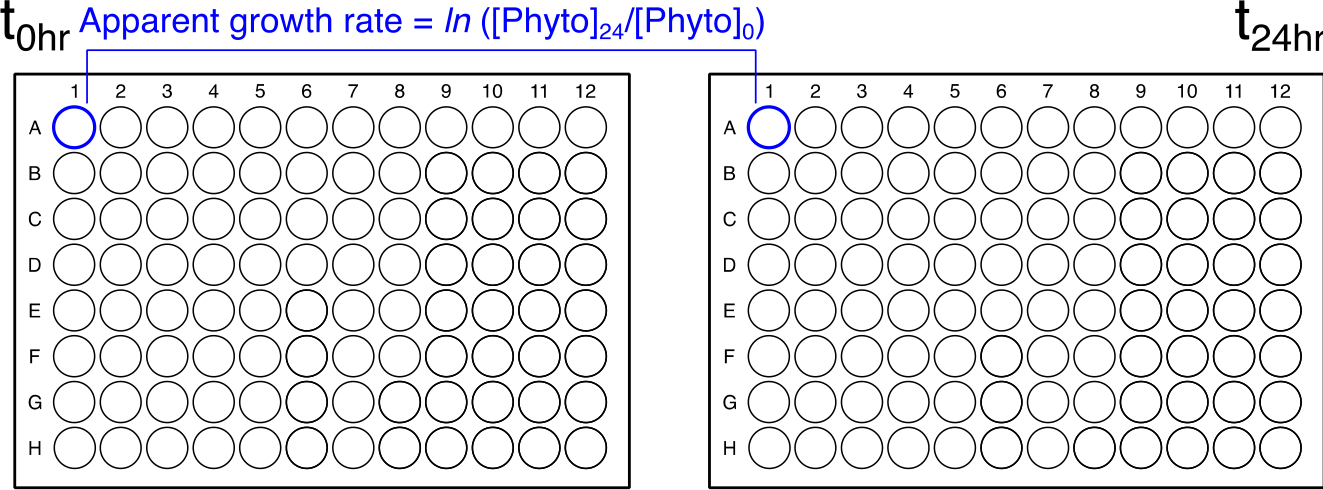
Supplementary Figure 8: Schematic of how rates were calculated from paired wells between the two plates.** The figure shows how the growth rate for the same in well A1 was calculated by pairing phytoplankton densities measured in well A1 at _t0_ and _t24_. The same was applied to all subsequent wells; well B1_t0_ values were paired with B1_t24_ values, C1_t0_ with C1_t24_, *etc.*

**Supplementary Figure 9:** **Cells counted in all experiments, dilution levels, and time points with associated coefficients of variation for initial counts.** Cells counted at the start of experiments (*t*_0_; *x*-axis; log_10_-transformed) are plotted against the relevent final counts (*t*_24_; *y*-axis; log_10_-transformed) across all dilution levels (87.5 % diluent, 75 % diluent, 50 % diluent, and undiluted 0 % diluent) for all experiments (columns; each experiment indicated by the letters in the grey boxes at the top of each column). Data points are color-coded by whether nutrients were added or not; nutrient unamended data are shown as black data points in the left panel and nutrient-added data as red data points in the right panel. Coefficients of variation for *t*_0_ values are shown in each plot. Note that coefficients of variation are the variance as a percentage of the mean for a given data set, calculated without log-transforming the data.
